# Impact of myocardial reperfusion on human plasma lipidome

**DOI:** 10.1016/j.isci.2022.103828

**Published:** 2022-01-29

**Authors:** Arun Surendran, Negar Atefi, Umar Ismail, Ashish Shah, Amir Ravandi

**Affiliations:** 1 Cardiovascular Lipidomics Laboratory, St. Boniface Hospital, Albrechtsen Research Centre, 351 Tache Avenue, Winnipeg, MB R2H 2A6, Canada; 2 Mass Spectrometry and Proteomics Core Facility, Rajiv Gandhi Centre for Biotechnology, Thiruvananthapuram, 695014 Kerala, India; 3 Department of Physiology and Pathophysiology, Rady Faculty of Health Sciences, University of Manitoba, 66 Chancellors Cir, Winnipeg, MB R3T 2N2, Canada; 4 Section of Cardiology, Department of Medicine, Rady Faculty of Health Sciences, University of Manitoba, 66 Chancellors Cir, Winnipeg, MB R3T 2N2, Canada

**Keywords:** Cardiovascular medicine, Lipidomics

## Abstract

The primary aim of the study is to investigate the temporal changes in plasma lipidome before and after reperfusion in patients with ST-segment elevation myocardial infarction (STEMI) and their association with myocardial injury. We found that 56% of the identified lipid species were significantly altered (corrected p< 0.05) in the first 24 h following reperfusion in patients with STEMI. Three lipid species, namely, acylcarnitine 18:2, TG 51:0, and LPC 17:1 were associated with a change in troponin concentration (delta troponin) and in-hospital cardiovascular events. Of these, acylcarnitine 18:2, and LPC 17:1 and their respective whole class levels, were significantly higher (p < 0.05) in the STEMI population than the age/sex-matched control subjects. Overall, our analyses showed a large shift in plasma lipidome in patients that undergo myocardial reperfusion. The differences found for acylcarnitines and LPC species and their association with both cardiac markers and cardiac outcomes need further validation.

## Highlights

• Human plasma lipidome rapidly shifts during myocardial reperfusion injury
• Novel plasma lipids are associated with cardiovascular events
• Acylcarnitines and lysoPCs correlate with the extent of myocardial injury
• Acute MI results in elevated plasma AC 18:2 and LPC 17:1 compared to controls

## Graphical abstract

**Figure:**
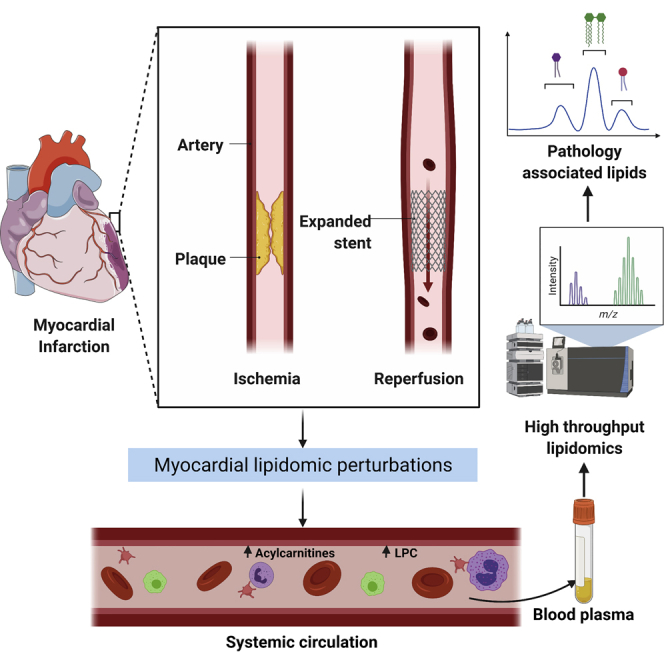

## Introduction

Cardiovascular diseases (CVDs) accounted for ≈18.6 million deaths in 2019, 32% of all deaths globally (Virani et al., 2021). Nearly 85% of these deaths were attributed to acute myocardial infarction (MI) and stroke (World Health Organization, 2021). After an acute MI, rapid coronary reperfusion using primary percutaneous coronary intervention (PCI), or intravenous fibrinolytic therapy is essential to restore blood flow and limit infarct size. Paradoxically, however, these treatment strategies can, in itself, induce additional myocardial injury, termed reperfusion injury, for which there are still no effective treatment options available (Hausenloy and Yellon, 2013). Experimental models of myocardial ischemia and infarction suggest that reperfusion injury contributes nearly 50% of the final infarct size (Yellon and Hausenloy, 2007). The biggest impact of ischemia/reperfusion (IR) injury in clinical settings arises in patients with ST-segment elevation (STEMI). Even after timely reperfusion, patients with reperfused STEMI have a 30-day mortality rate of nearly 5% (Lambert et al., 2010).

The conventional measurements for evaluating CVD risk include circulating lipid biomarkers such as cholesterol levels, triglycerides, high-density lipoprotein (HDL), and low-density lipoprotein (LDL). However, these measurements provide limited information on the individual lipid species and their association with the disease. Human plasma lipidome in a tightly regulated environment, and its composition can reflect the underlying phenotype in health and disease states (Burla et al., 2018a). Latest developments in high-throughput liquid chromatography and mass spectrometry have enabled us to identify associations between plasma lipid species and disease states. In a large population-based study, Alshehry et al. identified 32 plasma lipids significantly associated with cardiovascular events and death (Alshehry et al., 2016). Stegemann and colleagues showed that circulating lipid species outperform conventional lipid measures in CVD risk prediction (Stegemann et al., 2014). Meikle et al. demonstrated the potential of plasma lipid profiling for the risk stratification of unstable coronary artery disease (CAD) (Meikle et al., 2011). From all these results, it is now becoming more apparent that individual lipid species and classes can better reflect CVD risk and underlying pathophysiology than traditional lipid biomarkers.

Inflammation and oxidative stress accompanied by altered lipid metabolism are the prime drivers for the irreversible myocardial damage following ischemia and reperfusion (Yellon and Hausenloy, 2007; Yeang et al., 2019; Solati et al., 2018; Bilsen et al., 1989). However, little information is available about the changes in plasma lipidome in the setting of human myocardial IR injury. To better understand lipid biology during IR, it is necessary to assess lipid changes as a whole and not in individual parts accommodating both early and late reperfusion phases. Our recent non-targeted metabolomics study on patients with STEMI showed that lipids formed the bulk of the altered plasma metabolome following reperfusion. Also, we identified specific lipid species that were highly correlative with the extent of myocardial injury (Surendran et al., 2019). In this report, we aim to investigate the changes in circulating plasma molecular lipids in the setting of a multifactorial process such as human myocardial reperfusion injury.

## Results

### Patient characteristics

Table 1 summarizes the clinical profile of the study participants. Overall, 67% of the STEMI cohort were male with an average age of 64 years (63.71 ± 12.21). The STEMI and control group participants were matched for conventional cardiovascular risk factors, including age, gender, and BMI, apart from smoking status and dyslipidemia. The medication use was also similar between the two groups. Heparin was administered intravenously before the coronary angiography procedure in all participants, including the control subjects. In our STEMI cohort, 36% had occlusion in the left anterior descending (LAD) coronary artery, 37% had occlusion in the right coronary artery (RCA), and 10% had occlusion in the circumflex coronary artery.

**Table 1:** Baseline characteristics of the study participants

	Control (n = 50)	STEMI (n = 80)	*p* Value
Age (years)	60.92 ± 10.72	63.71 ± 12.21	0.187
Male sex (%)	31 (62.0)	54 (67.5)	0.521
LVEF (%)	60 (58, 60)	60 (45, 70)	0.679
Body mass index (kg/m^2^)	28.85 (26.17, 32.37)	27.67 (23.56, 32.40)	0.190

**Comorbidity** (%)			

Hypertension	23 (46.9)	37 (46.2)	0.939
Diabetes mellitus	7 (14.0)	16 (20.0)	0.383
Current smoker	6 (12.0)	22 (27.5)	0.036
Dyslipidemia	13 (26.0)	42 (52.5)	0.003
Hx of CAD	2 (4.0)	11 (13.8)	0.071

**Laboratory data**			

Triglyceride (mmol/L)	1.5 (1.0, 2.3)	1.3 (1.0, 2.1)	0.355
Cholesterol (mmol/L)	4.5 (3.9, 5.1)	4.8 (4.1, 5.3)	0.503
HDL cholesterol (mmol/L)	1.29 (0.9, 1.7)	1.10 (0.9, 1.37)	0.093
LDL cholesterol (mmol/L)	2.80 (1.93, 3.30)	2.80 (1.95, 3.45)	0.851
Creatinine (mmol/L)	78 (75, 84)	89 (71, 105.75)	0.086

**Medications at baseline (%)**			

ASA	8 (16.0)	18 (22.5)	0.367
ACEI/ARB	10 (20.0)	20 (25.0)	0.510
Beta blocker	9 (18.0)	8 (10.0)	0.188
Statin	8 (16.0)	20 (25.0)	0.225

**Additional parameters**			

Minutes from the onset of chest pain to reperfusion	NA	149.5 (86.25, 246.25)	
Peak CK (Units/L)	NA	975 (372, 2335)	
Peak TnT (ng/L)	NA	2029 (1102, 5943.50)	

**Culprit vessel (%)**			

LAD Infarct (%)	NA	36 (45.0)	
RCA Infarct (%)	NA	37 (46.2)	
Circumflex Infarct (%)	NA	10 (12.5)	

Values are reported as mean ± SD (SD), median (25th, 75th percentiles), or count (percentage) as applicable. The Chi-square test was used for categorical variables, while Student’s t-test or Mann-Whitney U test was used for continuous variables to assess for statistical significance across sample groups as applicable based on data distribution. Abbreviations: LVEF = left ventricular ejection fraction; Hx of CAD = history of coronary artery disease; HDL = high-density lipoprotein; LDL = low-density lipoprotein; ASA = Acetylsalicylic acid; ACEI = ACE (ACE) inhibitors; ARB = Angiotensin II receptor blockers; CK = Creatine kinase; TnT = troponin T; LAD = Left anterior descending coronary artery; RCA = Right coronary artery.

### Time course of the plasma lipidome

**Figure 1:**
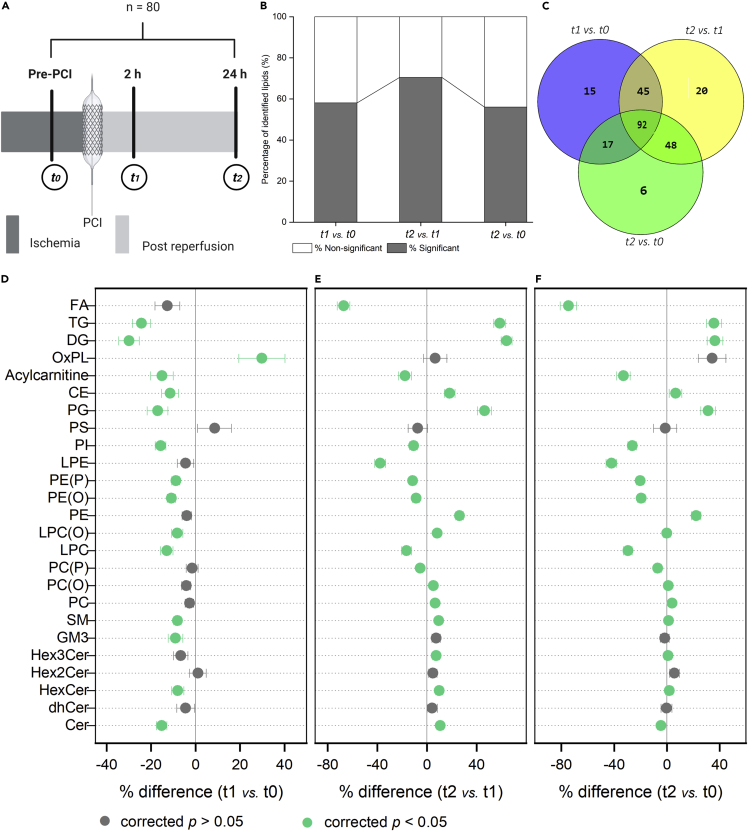
Temporal changes in plasma lipidome (A) Study design: The samples were collected by venipuncture at three different time points; the first, after STEMI diagnosis but before primary PCI (t0); the second, 2 h post-PCI (t1) and the third, 24 h post-PCI(t2). (B) Percentage of statistically significant lipids (corrected p< 0.05) at different time points. (C) Venn diagram showing the number of lipids shared or unique at different time points. (D–F) Forest plots showing total lipids expressed as percentage difference comparing each time point to one another. Data are represented as mean ± SEM. The corrected p values in (B), and (D)–(F) are obtained by repeated measures ANOVA followed by pairwise comparisons after Bonferroni correction. Gray circles represent the non-significant lipid classes/subclasses, and green circles represent the classes/subclasses with corrected p< 0.05. Table S1 contains the list of lipid abbreviations.

First, to investigate the temporal changes in lipidome after PCI, lipid profiling was performed on plasma from patients with STEMI at three-time points (t0, t1, and t2). Figure 1A illustrates the study design. Overall, plasma lipidome was markedly altered in patients with STEMI in the first 24 h after PCI. In the acute phase of reperfusion (t1 vs. t0), 58% of the identified lipids were significantly altered (corrected p< 0.05), which increased to 70% with the increase in reperfusion time (t2 vs. t1) (Figure 1B). In total, 56% of the identified lipid species were altered in the first 24 h after PCI (t2 vs. t0). The Venn diagram (Figure 1C) shows that a panel of 92 lipids was significantly altered (corrected p< 0.05) across all three-time points. Also, each time point is characterized by its distinctive lipidomic feature (Table S5). Six lipids, namely PC(O-36:3), CE 22:5, 16:0 acylcarnitine, 20:0 acylcarnitine, PGPC, and PONPC, signify the unique lipidomic difference between pre-PCI and 24 h post-PCI (t2 vs. t0).

Figures 1D–1F highlights the differences in the whole lipid class amount at each interval. The lipid data are shown as a percentage difference in total lipids compared to other time points (Tables S6–S8). Compared with pre-PCI levels, the majority of lipid classes/subclasses (15 out of 25) were significantly lower (corrected p< 0.05) in the acute reperfusion phase (t1 vs. t0) (Figure 1D). The only exception was the total oxidized phospholipid (OxPL) amount, whose levels increased significantly (29.87% higher, corrected p = 0.026) after PCI treatment. Total DG and TG also displayed large differences in the acute reperfusion phase. Their levels were 29.92 and 24.28% lower at 2 h post-PCI relative to pre-PCI (corrected p = 5.2 × 10^−^^8^, 2.91 × 10^−^^7^, respectively). However, total DG and TG exhibited a reverse trend over the next 24 h (t2 vs. t1). Their levels increased by 64.06 and 58.62% (corrected p = 1.38 × 10^−^^21^, 3.96 × 10^−^^18^, respectively) at 24 h post-PCI relative to 2 h post-PCI (Figure 1E). Overall, between pre-PCI and 24 h post-PCI (Figure 1F), the total amount of circulating fatty acids (FAs) showed the most notable difference (t2 vs. t0). They were 74.5% lower in pre-PCI than 24 h post-PCI (corrected p = 4.49 × 10^−18^).

### Perturbations in molecular lipid species

**Figure 2:**
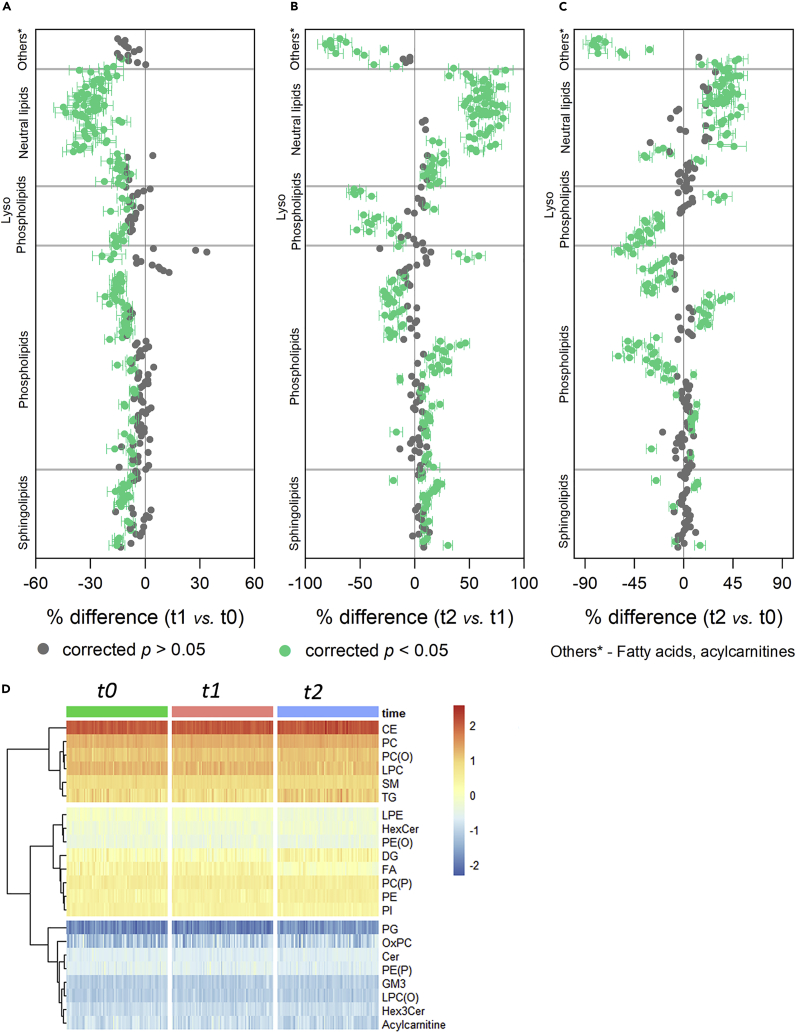
Perturbations in individual lipid species (A–C) Forest plot showing individual lipid species expressed as percentage difference comparing each time point to one another. Data are represented as mean ± SEM. The corrected p values are obtained by repeated measures ANOVA followed by pairwise comparisons after Bonferroni correction. Gray circles represent the non-significant lipid species, and green circles represent the lipid species with corrected p< 0.05. (D) Hierarchical heatmap showing the abundance of the detected lipid species across different sampling time points. Color code indicates lipid concentration (log-transformed).

Next, to attain a more in-depth view of the species level, forest plots were employed to visualize the change in individual lipid species at each time point. Consistent with the analysis of whole class amount, the majority of the individual lipid species were significantly lower (corrected p< 0.05) in the acute reperfusion phase compared with pre-PCI levels (Figure 2A and Tables S9–S11). On the contrary, the levels of two OxPL species, namely, PGPC and PONPC, were 33.85 and 27.74% higher at 2 h post-PCI than pre-PCI. During this period (t1 vs. t0), the most remarkable change was observed for neutral lipid species, including DG and TG. On average, they were 30% lower at 2 h post-PCI than pre-PCI. However, over the next 24 h (t2 vs. t1), these neutral lipids shifted in the opposite direction (Figure 2B). Their mean values at 24 h post-PCI were around 58% higher than 2 h post-PCI. Overall, FA species displayed the greatest change in the first 24 h after reperfusion (t2 vs. t0). On average, the levels of each FA species at 24 h post-PCI were about 69% lower than pre-PCI (Figure 2C).

The hierarchical heatmap (Figure 2D) illustrates the similarities and disparities in lipid concentrations across different times. At the lipid class level, there is little difference in lipid concentrations across various time points. However, along the heatmap rows, three major clusters based on plasma lipid abundance can be identified. By lipid class, the top-5 lipid concentrations followed the next order: CE > PC > PC(O) > LPC > SM. Conversely, the bottom-5 least concentrated lipid classes in plasma were acylcarnitine, Hex3Cer, LPC(O), GM3, and PE(P). This ranking in lipid concentration remained unaffected before and after reperfusion.

### Changes in lipidome by fatty acyl chain length and double-bond content

**Figure 3:**
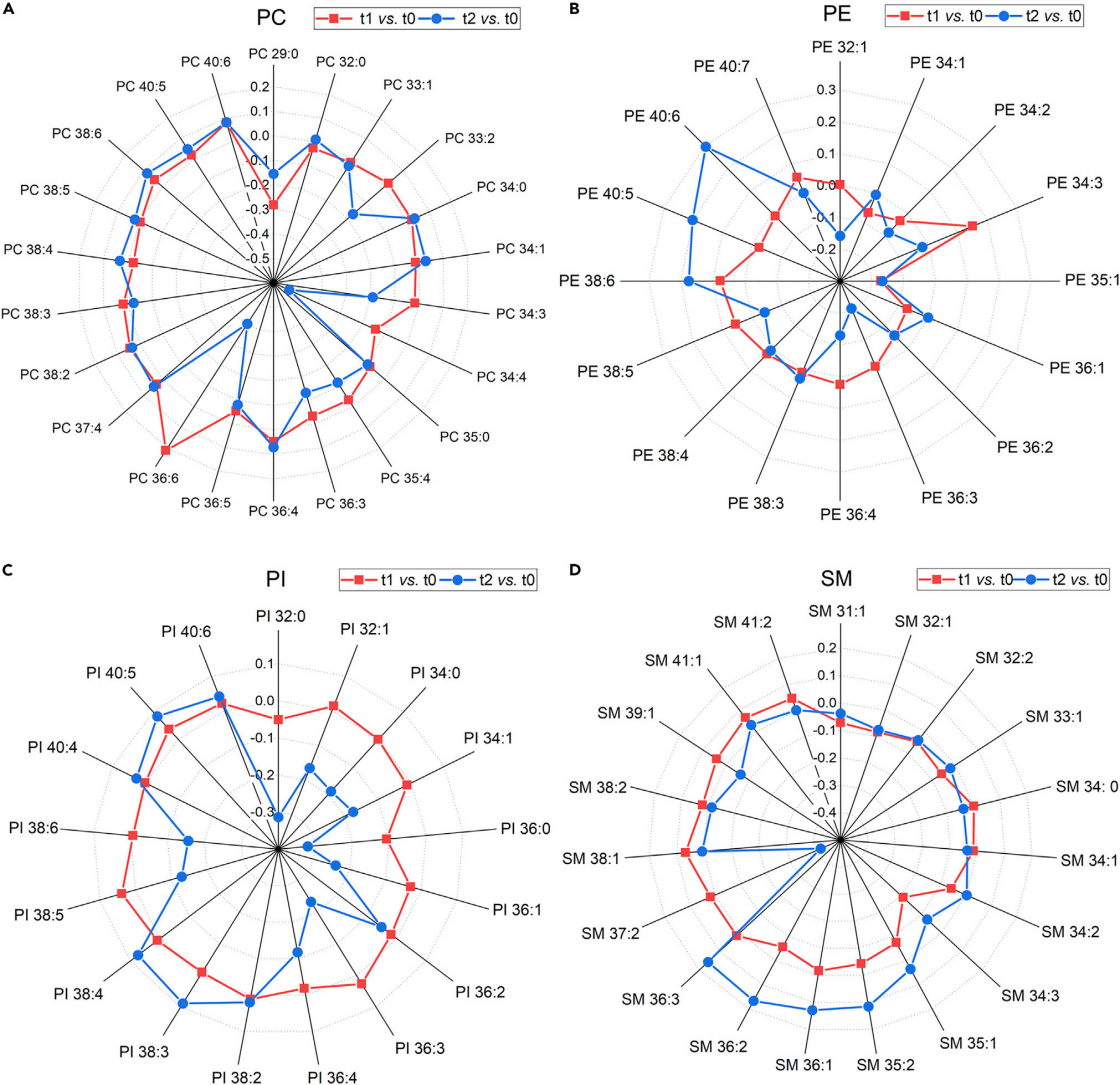
Alteration in lipid signal by the total number of carbon atoms and the degree of acyl chain saturation (A–D) The relative concentration of lipids was expressed as the concentration of lipids per sample to the total abundance within (A) phosphatidylcholine, (B) phosphatidylethanolamine, (C) phosphatidylinositol, and (D) sphingomyelin classes. The red color indicates the log ratio of the relative concentration at 2 h post-PCI (t1) compared to pre-PCI (t0). The blue color indicates the log ratio of the relative concentration at 24 h post PCI (t2) compared to pre-PCI (t0). Abbreviations: PC, phosphatidylcholine; PE, phosphatidylethanolamine; PI, phosphatidylinositol; SM, sphingomyelin.

Next, we investigated whether there was any pattern in changes in lipid signals according to carbon atom numbers and degree of unsaturation (number of double bonds) before and after reperfusion. We focused our attention on major structural lipids, namely PC, PE, PI, and SM. Figure 3A–3D demonstrates the relative concentration of lipid species at each time (t1, t2) compared to pre-PCI levels (t0). The values were normalized per sample to the total abundance within a lipid class. Based on the pattern of the number of carbon atoms and unsaturation degree, there was no alteration in concentration ratio in most of the PC species (Figure 3A) except for PC 36:6 and PC 34:4. These two species with higher double bond numbers were highly altered following reperfusion. Among the PE species, two 40 carbon atoms species, PE 40:5, PE 40:6, characterized by a high degree of unsaturation, exhibited the most remarkable change (Figure 3B). Among the PI class, almost all species were altered in both directions (Figure 3C). The most considerable change within the SM class was observed for the odd-numbered carbon atom with a double bond, namely SM 37:2 (Figure 3D). Also, SM species with relatively low carbon atom numbers (31, 32, 33, and 34) were not altered at different sampling time points (t0, t1, t2).

### Lipid signatures specific to ischemia/reperfusion injury

**Figure 4:**
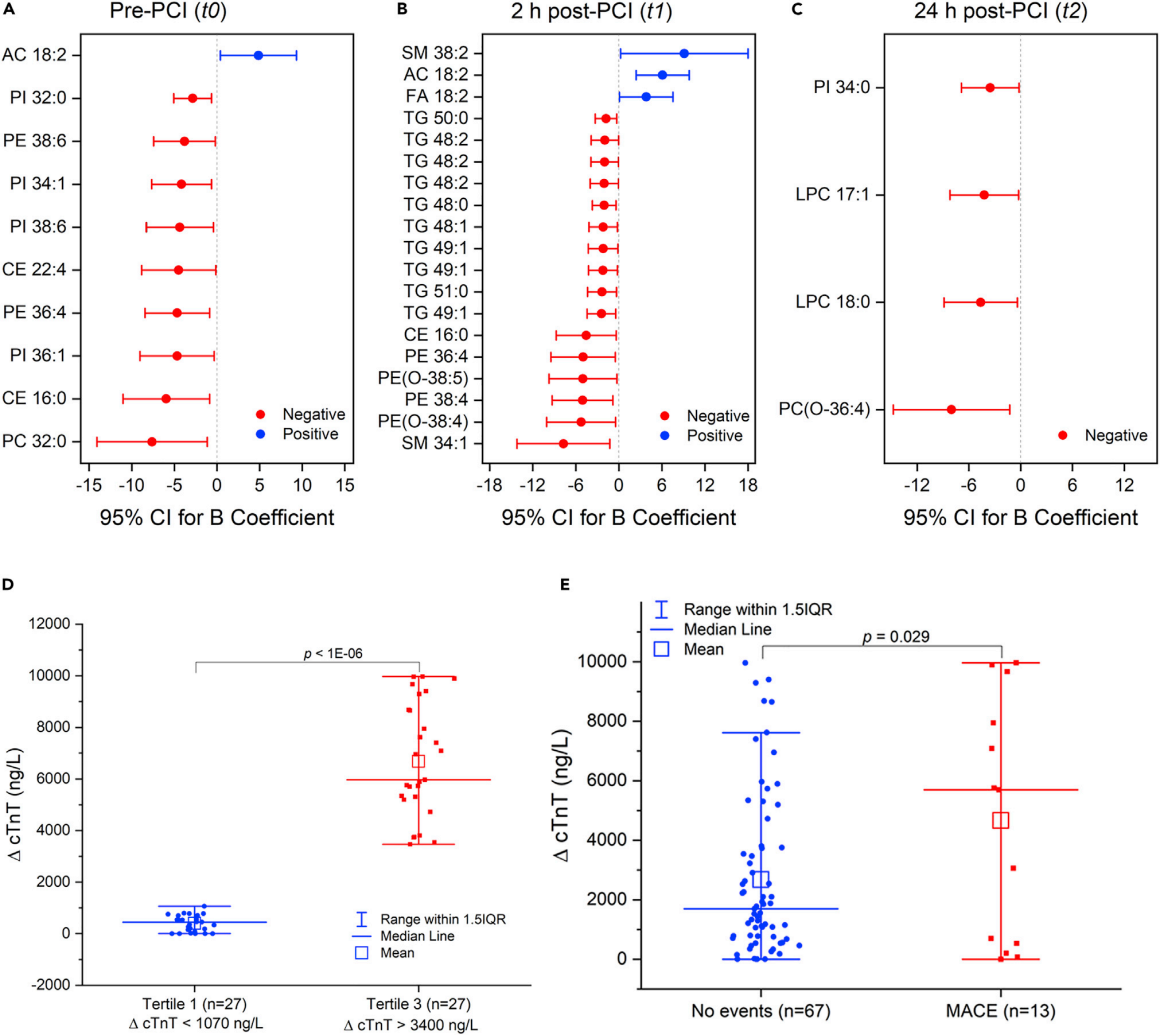
Association between delta troponin and plasma lipid species Linear regression analysis between delta troponin and log-transformed concentrations of each lipid species was performed after adjusting for age, sex, body mass index (BMI), current smoking, diabetes history, and time from symptom onset to reperfusion. The unit of delta troponin is mg/L. The blue color shows significant positive correlations (p< 0.05), and the red color shows significant negative correlations (p< 0.05). Whiskers represent 95% CIs (CI). (D) Delta troponin (Δ cTnT) amount in top (n = 27) and combined middle and bottom tertiles (n = 53). (E) Delta troponin (Δ cTnT) amount in MACE (n = 13) and “No events” (n = 67) groups. p values in (D) and (E) are by Student’s unpaired t-test. Abbreviations: B, Unstandardized B Coefficient; MACE, Major Adverse Cardiovascular Events; AC, Acylcarnitine.

Elevated cardiac troponin levels after PCI correlate with myocardial necrosis (Selvanayagam et al., 2005) and are also associated with worse 90-day clinical outcomes (Cantor et al., 2002). In our STEMI cohort, cardiac troponin T (cTnT) was measured daily for the first 72 h at regular intervals. Here, the delta troponin (Δ cTnT) value for each patient is defined as the absolute change in baseline (pre-PCI) troponin value from peak troponin value. A linear regression of delta troponin against lipid species was performed after adjusting for age, sex, body mass index (BMI), current smoking, diabetes history, and ischemic time (time from symptom onset to reperfusion) to investigate lipid changes specific to reperfusion injury (Figures 4A–4C, Tables S12–S14). After the adjustment of these major cardiovascular risk factors, ten lipid species were significantly associated (p< 0.05) with delta troponin at pre-PCI (t0), 19 lipid species were significantly associated with delta troponin at 2 h post-PCI (t1), and four lipid species were significantly associated with delta troponin at 24 h post-PCI (t2). Notably, all the significant lipid associations at pre-PCI (t0) were negative except for acylcarnitine 18:2, which was positively associated with delta troponin (Figure 4A). Similarly, except for three lipid species, namely acylcarnitine 18:2, FA 18:2, and SM 38:2, all other lipids were significantly negatively associated with delta troponin at 2 h post-PCI (Figure 4B). These included mainly TG, PE, and PE(O) classes. Only four lipid species (Figure 4C) were associated with delta troponin at 24 h post-PCI (t2), and among them, two were LPC species (LPC 17:1 and LPC 18:0). In total, 33 lipids were identified, which significantly associate (p< 0.05) with delta troponin across three-time points.

### Lipids and the severity of myocardial ischemia/reperfusion injury

**Figure 5:**
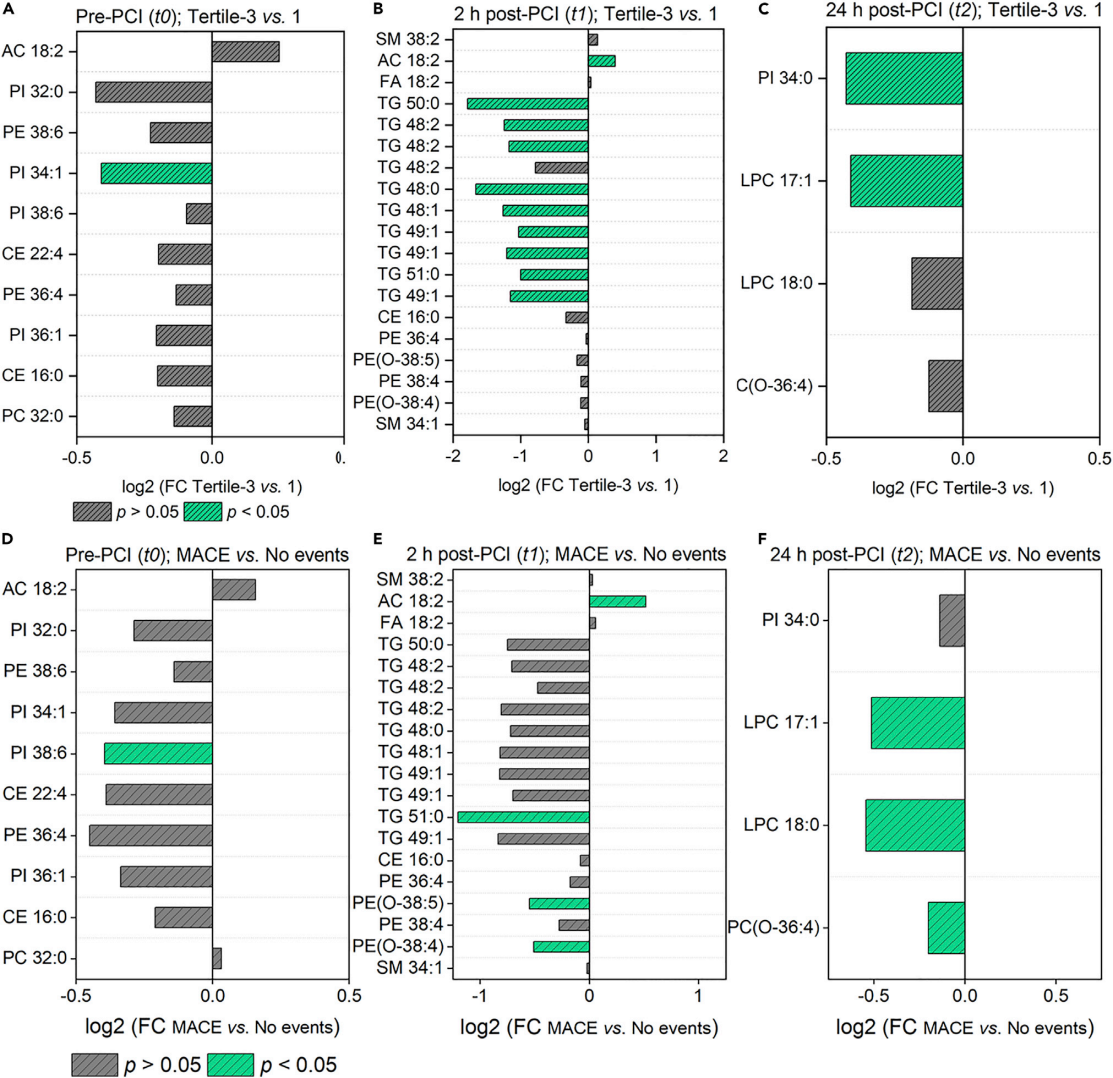
Lipid species and the extent of myocardial injury (A–C) Log2 fold change of lipid abundance in top tertile (Tertile-3, n = 27) compared to lower tertiles (Tertile-1&2, n = 53) across different time points. (D–F) Log2 fold change of lipid abundance in MACE group (n = 13) compared to “No events” group (n = 67) across different time points. The gray color represents the non-significant lipid species, and the green color represents the significant (p< 0.05) lipid species. p values are by Student’s unpaired t-test. Abbreviations: FC, fold change; AC, Acylcarnitine.

Next, we examined whether the delta troponin-associated lipids could determine the severity of myocardial injury in patients with STEMI. In this regard, firstly, we classified the patients with STEMI into two groups based on their delta troponin values (Figure 4D and Table S15). Figures 5A–5C compares the direction, magnitude, and statistical significance of delta troponin-associated lipids between the top tertile (Δ cTnT >3400 ng/L) and the bottom (lower) tertile (≤1070 ng/L). Among the ten delta troponin-associated lipids at pre-PCI, only one lipid - acylcarnitine 18:2 - was higher in the top tertile (Figure 5A). The rest of the lipids (9/10) were lower in abundance in the top tertile, and among them, PI 34:1 was significantly lower (p< 0.05) in the top tertile. Ten out of 19 delta troponin-associated lipids at 2 h post-PCI differed significantly between the two tertiles (Figure 5B). Of these 10 lipids, the level of acylcarnitine 18:2 was significantly higher (p< 0.05) in the top tertile. The rest, mainly comprising TG species, were significantly lower (p< 0.05) in the top tertile. Amongst the four delta troponin-associated lipids identified at 24 h post-PCI (Figure 5C), two of them, namely PI 34:0, and LPC 17:1, were significantly lower (p< 0.05) in the top tertile compared to the lower tertile. The significance of these lipids with delta troponin values holds true even after adjusting for differences in the HDL levels between the top and bottom tertiles (Table S16).

We then examined the role of these delta troponin-associated lipids in major adverse cardiovascular events (MACE). We calculated the incidences of MACE (cardiovascular death, re-hospitalization, MI, and congestive heart failure) in our STEMI cohort. Accordingly, patients with STEMI were categorized into the MACE group (n = 13) and the “No events” group (n = 67) (Table S17). As shown in Figure 4E, the delta troponin values differed significantly (p< 0.05) between the MACE and “No events” groups. Figures 5D–5F compares the direction, magnitude, and statistical significance of delta troponin-associated lipids between the two groups. Notably, except for one lipid– PC 32:0 – all other lipids followed the same direction (higher/lower) similar to the top vs. lower tertile comparison. Interestingly, two lipid species, namely acylcarnitine 18:2, and TG 51:0, which differed significantly (p< 0.05) between the top and lower tertile of delta troponin at 2 h post-PCI (Figure 5B), exhibited a similar significant distinction (p< 0.05) between MACE and the “No events” group at the same period (Figure 5E). Likewise, LPC 17:1 which differed significantly (p< 0.05) between the top and lower tertile of delta troponin at 24 h post-PCI (Figure 5C), followed the same trend in MACE vs. “No events” comparison (Figure 5F). The above-described analysis based on delta troponin tertiles and MACE groups demonstrated that three lipid species, namely acylcarnitine 18:2, TG 51:0, and LPC 17:1, significantly associate (p< 0.05) with delta troponin, and their levels differed significantly (p< 0.05) between patients with sizable myocardial injury and patients with lesser myocardial injury. The significance, timing, and direction of change in these cardiac-specific lipids remained the same in MACE vs. “No events” comparison as well.

### Early versus late phase of reperfusion

**Figure 6:**
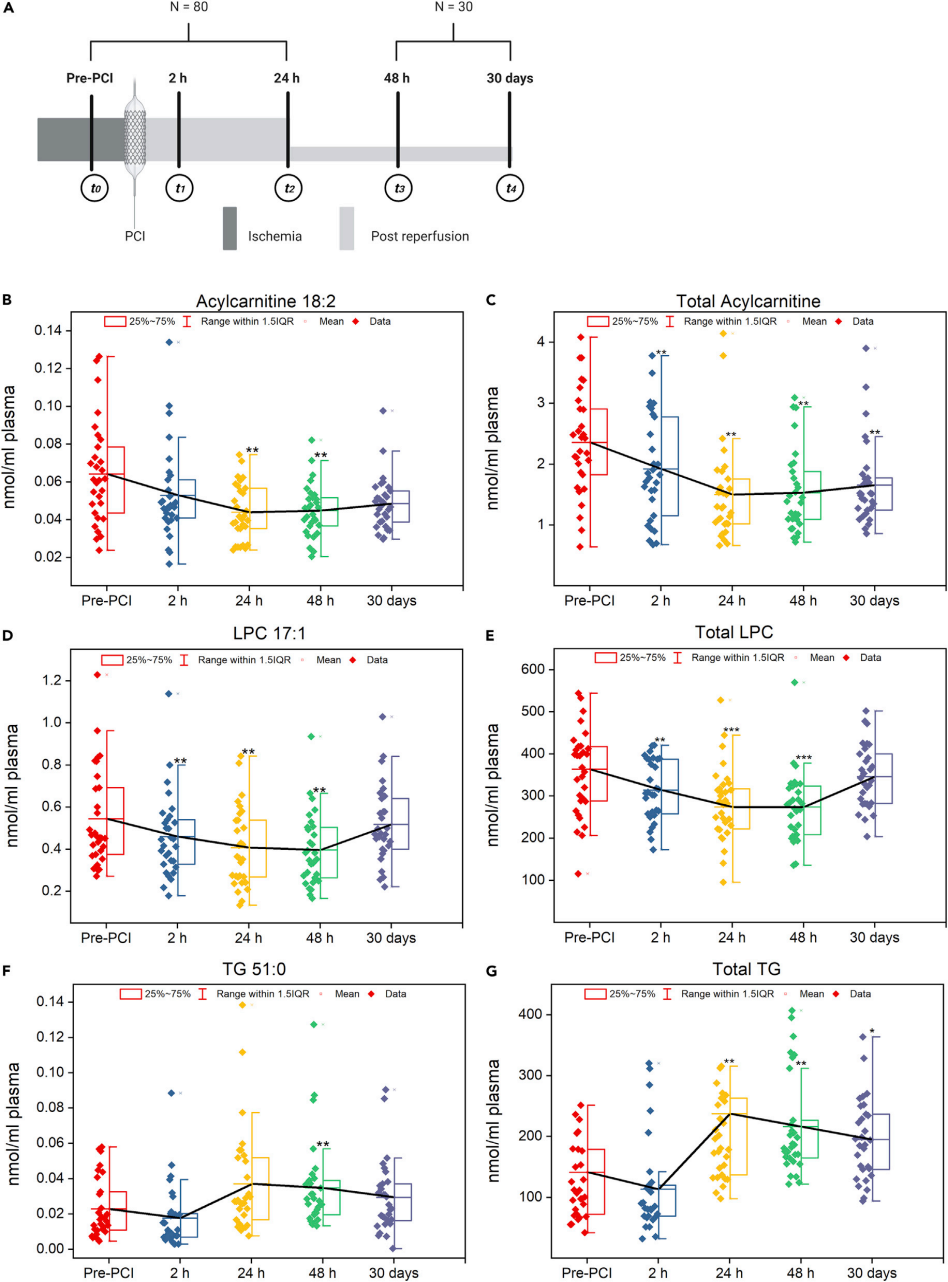
Lipid species at the late reperfusion phases (A) Study design: In a subset of the population (n = 30), the samples were collected by venipuncture at two additional time points; 48 h post- PCI (t3) and 30 days post-PCI (t4). (B–J) Distribution of specific lipid species and lipid classes in the early (pre-PCI, 2 h, 24 h) and late phases (48 h, 30 days) of reperfusion. The black line represents the mean of lipid species/classes. The p values were obtained by repeated measures ANOVA followed by pairwise comparisons after Bonferroni adjustment. Asterisks (∗, ∗∗ and ∗∗∗) indicate statistical significance at p<0.05, p< 0.01, and p< 0.001, respectively, to the baseline (pre-PCI).

We prospectively followed a subset of our cohort (n = 30) for another 30 days (Figure 6A) to detect the changes in the three cardiac-specific lipids from early to late reperfusion. We observed that specific patterns characterize these lipids during early and late reperfusion phases. Except TG 51:0 and total TG, the levels of these cardiac-specific lipid species (acylcarnitine 18:2, LPC 17:1) as well as their respective whole class amount, including total acylcarnitine, and total LPC, progressively decreased up to 48 h after PCI before climbing again (Figures 6B–6E). Though levels of TG 51:0 and total TG decreased immediately after PCI (Figures 6F–6G), their levels sharply rose after 2 h of reperfusion, reached the maximum over the next 24 h (24 h post-PCI), and then plummeted again in the late phases of reperfusion (48 h and 30 days post-PCI). In short, the plasma lipid levels of acylcarnitine 18:2, LPC 17:1, and TG 51:0 as well as their respective whole class amount differed significantly (p< 0.05) at late reperfusion phase (48 h post-PCI) compared with pre- PCI.

### Comparison with controls subjects

**Figure 7:**
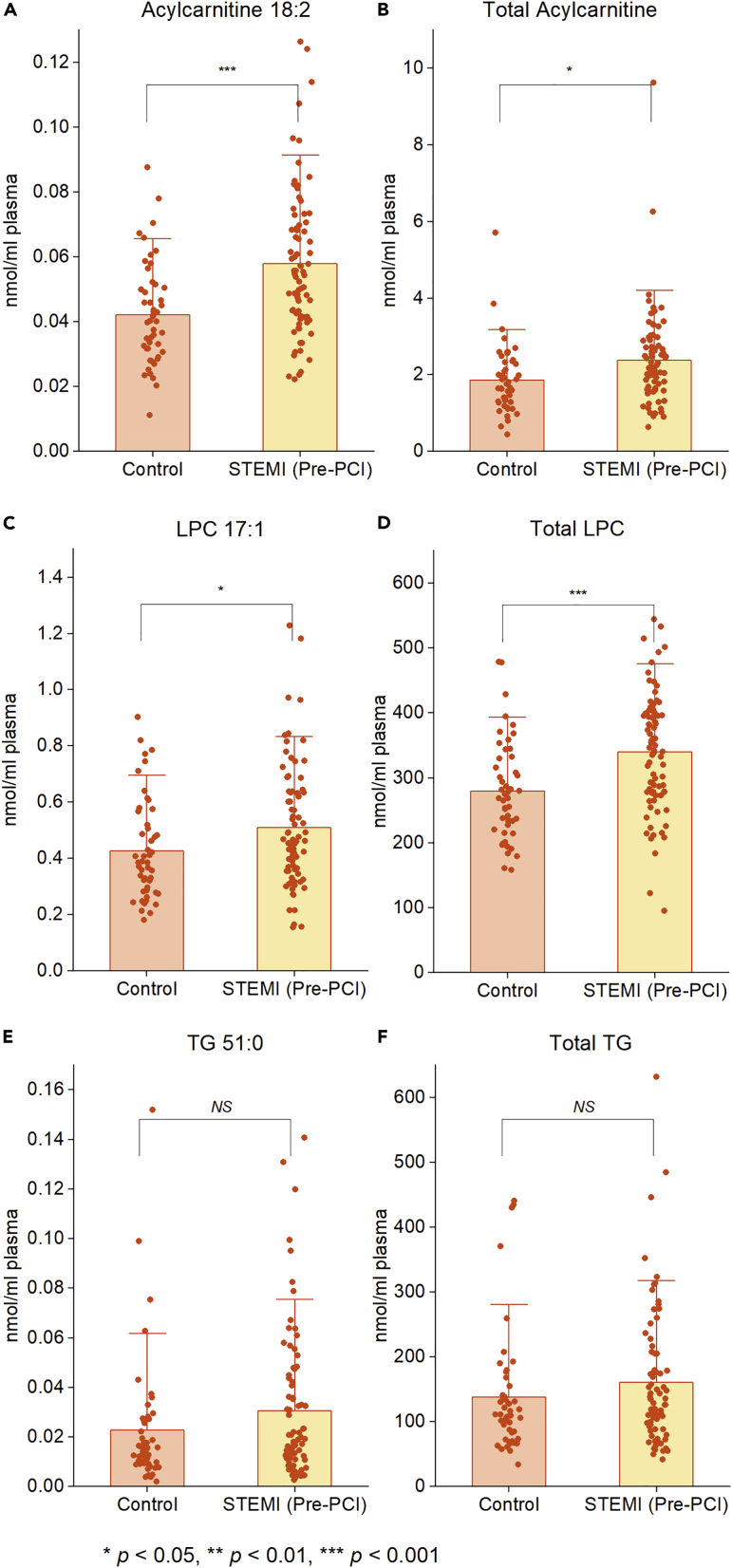
Significantly perturbed lipid species identified in patients with STEMI compared to controls (A–F) Distribution of specific lipid species and lipid classes among the STEMI (pre-PCI) and control groups. p values are by Student’s unpaired t-test. Asterisks (∗, ∗∗ and ∗∗∗) indicate statistical significance at p< 0.05, p< 0.01, and p< 0.001, respectively, after Student’s t-test.

Next, we examined whether the concentration of these cardiac-related lipid species/classes in patients with STEMI at the time of presentation (pre-PCI) is comparable with that of an age and gender-matched control population without any intervention. Based on Student’s unpaired t-test, the levels of acylcarnitine 18:2 and total acylcarnitine were significantly higher (p< 0.05) in the STEMI group relative to the control subjects (Figures 7A–7B). Likewise, the concentration of individual LPC 17:1, as well as the total LPC amount, were also significantly higher (p< 0.05) in the STEMI group relative to the control subjects (Figures 7C–7D). No significant differences were noted for TG 51:0, and its respective total class amount between the two groups (Figures 7E–7F).

## Discussion

This study elucidated a detailed lipidomic map of human plasma during myocardial ischemia and reperfusion. Given that there are currently no clinical therapies for reperfusion injury, we sought to understand the temporal changes in plasma lipids before and after reperfusion and their correlation with clinical markers of cardiac injury. The vast majority of research in the pathophysiology of IR injury has focused on the genetic and protein expression changes during reperfusion, with little to no attention given to the sizable myocardial lipid pool that harbors profound biological activity. This may be why there are still no proven therapeutic options that allow for lowering the cardiotoxic effects of reperfusion injury. Our study is unique as it allows for the determination of the dynamic changes in circulating lipidome as a whole before and after reperfusion and its association with myocardial reperfusion injury. The IR injury not only affects the ischemic organ alone (here, heart) but also may also cause systemic damage to other organs, and can possibly lead to multiorgan dysfunction. Therefore, we have used cardiac biomarkers such as troponin and cardiovascular outcomes to select cardiac-specific lipid molecules that are important during reperfusion and are less impacted by non-cardiac factors. Also, we employed a repeated measure study design in which the lipid values were measured on the same subject serially over time. In repeated measures designs, each subject serves as their own control. This helps control factors that cause variability between subjects, including diet and medications, and helps focus more precisely on the lipidomic changes owing to intervention (PCI).

The most striking observation in this study was that myocardial ischemia followed by reperfusion results in significant shifts within the plasma lipidome. We found that 56% of the circulating lipid species were significantly altered (corrected p< 0.05) in the first 24 h after PCI in patients with STEMI. In the acute phase of reperfusion (t1 vs. t0), there was an overall decrease in the majority of lipid classes/subclasses, including phospholipids. In line with this notion is the observation that in animal models of cerebral IR, there is a significant reduction in blood plasma phospholipids during reperfusion (Drgová et al., 2004). The authors attributed it to oxidative stress and activation of phospholipases following reperfusion. In a study to examine the effects of IR on rat hearts, Paradies et al. demonstrated a similar reduction in plasma lipids. They ascribed it, in part, to lipid peroxidation of unsaturated FAs by oxygen-free radicals (Paradies et al., 1999). The lone lipid class in our study that was significantly increased (corrected p< 0.05) immediately after reperfusion was oxidized phospholipids (OxPL). In an *in vivo* model of renal IR injury, we previously showed that OxPL species are produced in kidney tissue during renal IR injury. Their levels increase with the increase in reperfusion time (Solati et al., 2018). In patients with stable angina pectoris, Tsimikas and colleagues (Tsimikas et al., 2004) showed that circulating OxPL levels were elevated immediately after PCI. OxPLs have been recognized as toxic oxidative by-products of oxidative damage derived from sources of cellular injury such as plaque disruption and myocyte death (Yeang et al., 2019). The increased level of OxPL after reperfusion may reflect the increased oxidative stress immediately after reperfusion.

At the class level, the most substantial change in the first 24 h after PCI (t2 vs. t0) was observed for free FAs, followed by neutral lipids (DG, TG) and oxidized phospholipids. The increase in circulating neutral lipids after PCI coincided with the decrease in free FAs (Figures 1F and 2C). The same trend was observed for individual molecular species from these lipid classes as well. This indicates the presence of an active triglyceride-FA-triglyceride cycle during myocardial IR (Schoonderwoerd et al., 1989). Our findings also agree with the work by Saddik et al., who showed significant enhancement in triglyceride synthesis during reperfusion of ischemic rat hearts. They showed that despite an initial burst of FA β-oxidation, the rate of triglyceride lipolysis is not accelerated during reperfusion and was comparable to non-ischemic hearts (Saddik and Lopaschuk, 1992). This partly explains why triglyceride levels are not accompanied by a similar increase in circulating free FAs following reperfusion. Our results also showed that circulating FAs were elevated during ischemia (pre-PCI) compared to reperfusion (post-PCI). This observation is consistent with a recent study by Feng et al., which reported a decrease in circulating FAs with increased reperfusion time (Feng et al., 2018). The heart is the most metabolically active organ in the human body, and under normal conditions, free FAs are the primary energy source for cardiac ATP production. However, during myocardial ischemia, these FAs are detrimental both clinically and experimentally. The detrimental effects are mainly owing to the inhibition of myocardial glucose utilization and accumulation of toxic intermediates of FA metabolism, including long-chain acylcarnitines (Hendrickson et al., 1997; Lopaschuk et al., 1994). Mounting evidence suggests that the potential contributors to elevated levels of circulating FAs during myocardial ischemia are degradation of endogenous lipids such as triacylglycerols or phospholipids and release of stress hormones such as epinephrine and catecholamines (Saponaro et al., 2015; Saddik and Lopaschuk, 1992; Bilsen et al., 1989). After acute MI, the elevated free FA levels are of clinical importance related to reduced cardiac efficiency, increased risk of arrhythmias, and sudden death (Hendrickson et al., 1997; Saddik and Lopaschuk, 1992; Lopaschuk et al., 1994).

In most biological systems, the primary function of lipids is to serve as structural components of cell membranes. However, oxygen-free radicals produced during reperfusion can cause membrane damage and contribute to reperfusion injury (González-Montero et al., 2018). A comparison of saturation degree and chain length of FAs of major structural lipids (PC, PE, PI, and SM) before and after reperfusion can determine which lipid class is more prone to oxidation. Only a small number of PC species altered before and after reperfusion. Nevertheless, PE, PI, and SM showed great shifts between different time points during reperfusion (Figures 3A–3D). This suggests that PC species are likely more resistant to oxidative stress than other membrane phospholipids and offer resistance to damage in order to maintain membrane integrity (Piomelli et al., 2007). For PC species, the most extensive alteration was shown by 34–36 carbon atoms and four to six double bonds. For PE species, the most considerable alteration was shown by 40 carbon atoms and an unsaturation degree of 5–6. For PI species, all were altered; and for SM species, 37:2 showed the maximum diversity. We have previously shown that PI species are much more rapidly oxidized as LDL undergoes lipid oxidation. During 36 h of copper oxidation, there is a rapid loss of polyunsaturated PI species and the generation of oxidized PI species, which in terms of proportion are many folds higher than the generation of oxidized PC species within LDL (Hasanally et al., 2017). This is in proportion to PIs' large polyunsaturated content compared to PCs. In this study, PC and PE species with a medium-to-long acyl chain (34-40) and four, five, or six double bonds have shown alteration following reperfusion, likely as a result of susceptibility to oxidative stress.

Because of its high sensitivity and specificity, cardiac troponins are considered "gold standard" biomarkers for myocardial damage (Apple et al., 2017). In order to assess the ongoing myocardial tissue loss after reperfusion, we utilized the delta troponin post revascularization. This allowed us to determine the lipids that correlated with ongoing myocardial injury subsequent to revascularization. We found that 33 lipids associated with delta troponin at different time points (pre-PCI, 2h post-PCI, 24 h post-PCI) following adjustment for age, sex, BMI, current smoking status, diabetes history, and ischemic time (Figures 4A–4C). Three of these lipids, namely, acylcarnitine 18:2, TG 51:0, and LPC 17:1, could also stratify patients based on the severity of cardiac injury (Figures 5A–5C). They could also stratify those at increased risk of in-hospital cardiovascular events from regular ones (Figures 5D–5F). Also, their levels in the late reperfusion phase (48 h post-PCI) were significantly different (p< 0.05) from pre-PCI levels (Figures 6B–6G). In addition, among these cardiac-related lipids, the levels of acylcarnitine 18:2, and LPC 17:1 as well as their respective whole class amount were significantly higher (p< 0.05) in the STEMI population at the time of admission compared to the age and gender-matched control subjects (Figures 7A–7D). This points to the fact that not all cardiac-related lipids identified as significant during reperfusion can be used as biomarkers to distinguish control subjects from patients with STEMI. Of these, acylcarnitine 18:2 notably exhibited a positive association with delta troponin value at pre-PCI and 2 h post-PCI. Our previous non-targeted metabolomics study has shown that plasma levels of acylcarnitine 18:2 could determine the extent of cardiac injury in patients with STEMI (Surendran et al., 2019). The long-chain acylcarnitines are intermediates of FA metabolism (Adams et al., 2009). Their elevated circulating levels are widely used as diagnostic markers of FA oxidation disorders (McCoin et al., 2015). A growing corpus of literature highlights the impacts of long-chain acylcarnitines on various aspects of cardiovascular pathophysiology, such as cardiovascular death, acute MI, and stroke (Guasch-Ferré et al., 2016; Strand et al., 2017). In addition, it was shown that the inhibition of acylcarnitine accumulation could attenuate the incidence of lethal arrhythmias and contractile deficits following acute ischemia (Corr et al., 1989). Many previous studies revealed that saturated long-chain FAs activate some pattern recognition receptors (PRRs), such as toll-like receptor 2 (TLR2) and TLR4 (Lee et al., 2001, 2004). These findings result in the conclusion that acylcarnitines have the potential to mediate inflammation. Rutkowsky et al. later confirmed this finding and showed that medium and long-chain acylcarnitines activate pro-inflammatory signaling pathways, including PRRs (Rutkowsky et al., 2014). Our findings, therefore, fortify the role of acylcarnitines in cardiac injury and subsequent inflammation.

There are extensive *in vitro* and animal studies showing that reperfusion results in FA accumulation. However, when the TG levels increase owing to limited FA oxidation, as occurs in several pathological conditions, it can contribute to cardiac dysfunction and worsening contractile function (D'Souza et al., 2016). Even though both plasma CE and TG are predictors of the development of CAD and poor outcomes, there are conflicting reports on the changes of plasma TG during acute MI (Cheng et al., 2015) and specifically reperfusion. We identified ten triglyceride species with shorter carbon length and fewer double bonds (0,1,2) correlated with delta troponin at 2 h post-PCI. They were all negatively associated with delta troponin at 2 h post-PCI even after adjusting for clinical risk factors, including diabetes. TG 51:0 is also associated with the risk of adverse cardiovascular events. This finding agrees with prior studies demonstrating that specific triglycerides with a low carbon number and double-bond content were predictive of CVD (Stegemann et al., 2014; Toledo et al., 2017). The same triglyceride pattern was associated with BMI and abdominal adiposity in a metabolomics study among Framingham Offspring Cohort (Ho et al., 2016). Together, these findings suggest a relationship between triglycerides acyl chain content with cardiac injury in the setting of IR injury.

Two LPC species at 24 h post-PCI, namely LPC 17:1 and LPC 18:0, displayed negative associations (p< 0.05) with delta troponin. Supporting this observation, Meikle et al. have previously shown that LPC species containing saturated FAs were strongly associated with CAD (Meikle et al., 2011). LPCs are a well-recognized group of pro-inflammatory lipids (Law et al., 2019) whose levels in human circulation indicate atherosclerotic plaque inflammation and endothelial dysfunction (Lavi et al., 2007a; Law et al., 2019). Here, the levels of LPC 17:1, as well as the total LPC amount, were significantly higher (p< 0.05) in the STEMI population (pre-PCI) compared to control subjects. Elevated levels of LPC in circulation can result from the increased degradation of PC on lipoprotein particles via the action of enzymes such as lipoprotein-associated phospholipase A2 (Lp-PLA2) (Law et al., 2019; Packard et al., 2000). Lp-PLA2, itself is a marker of vascular inflammation (Lavi et al., 2007b) and suggests a pro-inflammatory role for LPC in the setting of IR injury.

### Limitations of the study

One limitation of our study is the small sample size. We have mitigated this by utilizing repeated measures analysis, which allows subjects to act as their control, mitigating the risk of confounding factors. The observed associations and relationships between cardiac-specific lipid markers in this article should be considered hypothesis-generating. Hence, further confirmatory studies in independent cohorts and preclinical and clinical models of myocardial ischemia and reperfusion are needed to understand better the mechanistic link between the lipids identified in this report and reperfusion injury.

### Conclusions

This study presents a detailed overview of the temporal changes in plasma lipidome in a clinical setting of myocardial reperfusion injury in humans. In short, reperfusion results in dramatic changes in the plasma lipidome. We identified significantly altered lipid classes/subclasses and lipid species before and after reperfusion. Although a small number of the identified lipids have been previously shown to associate with CAD (e.g., PE(O-38:5)), most lipid changes are novel in the setting of human I/R injury. We also identified three lipids, representing three lipid classes, associated with the severity of myocardial injury independent of other traditional CVD risk factors. These three lipids were also discriminatory for adverse cardiovascular events, suggesting their clinical relevance in the setting of IR injury, but further work is necessary to validate any of these findings.

## STAR★Methods

### Key resources table

**Table:** 

REAGENT or RESOURCE	SOURCE	IDENTIFIER
**Biological samples**		

Blood plasma	Homosapiens	N/A

**Chemicals, peptides, and recombinant proteins**		

LC/MS grade methanol	Fisher Chemical	Cat#A456-4
LC/MS grade tetrahydrofuran	Fisher Chemical	Cat#T425-4
LC/MS grade water	Fisher Chemical	Cat#W6-4
Analytical grade ammonium formate	Sigma-Aldrich	Cat#70221-25G-F
Analytical grade 1-butanol	Sigma-Aldrich	Cat#34867-1L
Analytical grade chloroform	Millipore Sigma	CAS-No:67-66-3
Dihydroceramide (dhCer 8:0)	Avanti Polar Lipids	Cat#860626P
Ceramide (Cer 17:0)	Avanti Polar Lipids	Cat#860517P
Sphingomyelin (SM 12:0)	Avanti Polar Lipids	Cat#860583P
Lysophosphatidylcholine (LPC 13:0)	Avanti Polar Lipids	Cat#855476C
Phosphatidylcholine (PC 13:0_13:0)	Avanti Polar Lipids	Cat#850340C
1,2-dinonanoyl-sn-glycero-3-phosphocholine (PC 9:0_9:0)	Avanti Polar Lipids	Cat#850320
Lysophosphatidylethanolamine (LPE 14:0)	Avanti Polar Lipids	Cat#856735P
Phosphatidylethanolamine (PE 17:0_17:0)	Avanti Polar Lipids	Avanti Polar Lipids
Phosphatidylglycerol (PG 17:0_17:0)	Avanti Polar Lipids	Cat#830456P
Phosphatidylserine (PS 17:0_17:0)	Avanti Polar Lipids	Cat#840028P
Acylcarnitine 3:0 (d5)	CDN Isotopes	Cat#D-7762
Acylcarnitine 14:0 (d3)	CDN Isotopes	Cat#D-6661
Cholesteryl ester (CE 18:0 (d6))	CDN Isotopes	Cat#D-5823
Fatty acid (FA 15:0 (d3))	CDN Isotopes	Cat#D-5258
Diacylglycerol (DG 15:0_15:0)	LGC Standards	Cat#LA 32-1503-8
Monohexosylceramide (HexCer 16:0 (d3))	MJS BioLynx	Cat#MT1533
Dihexosylceramide (Hex2Cer 16:0 (d3))	MJS BioLynx	Cat#MT1534
Trihexosylceramide (Hex3Cer 17:0)	MJS BioLynx	Cat#MT1523
Triacylglycerol (TG 17:0_17:0_17:0)	Sigma-Aldrich	Cat#T2151
1-palmitoyl-2- (5-oxovaleroyl)-sn-glycero-3-phosphocholine (POVPC)	Avanti Polar Lipids	Cat#870606P
1-palmitoyl-2-(9-oxo)nonanoyl-sn-glycero-3-phosphocholine (PONPC)	Avanti Polar Lipids	Cat#870605P
1-palmitoyl-2-glutaroyl-sn-glycero-3-phosphocholine (PGPC)	Avanti Polar Lipids	Cat#870602C
1-Palmitoyl-2-azelaoyl-sn-glycero-3-phosphocholine (PAzPC)	Avanti Polar Lipids	Cat#870600C
1-(palmitoyl)-2-(5-keto-6-octene-dioyl)-3-phosphocholine (KOdiAPC)	Cayman Chemicals	Cat#62945
1-palmitoyl-2-(4-keto-dodec-3-ene-dioyl)-sn-glycero-3-phosphocholine (KDdiAPC)	Cayman Chemicals	Cat#62935

**Deposited data**		

Raw data	Metabolights	MTBLS3839

**Software and algorithms**		

SPSS v24	IBM Corporation	N/A
R (version 3.5.2)	(R Core Team, 2020)	https://cran.r-project.org/

**Other**		

Zorbax C18, 1.8 μm, 50 × 2.1 mm column	Agilent Technologies	Cat#959757-902

### Resource availability

#### Lead contact

Further information and requests for resources should be directed to and will be fulfilled by the Lead Contact, Dr. Amir Ravandi (aravandi@sbgh.mb.ca).

#### Materials availability

This study did not generate new unique reagents.

### Experimental model and subject details

Venous blood samples were collected from 80 patients undergoing primary PCI for STEMI between January 2017 and June 2018. The diagnosis of STEMI was based on presentation with chest pain, confirmation of ST-elevation on 12-lead ECG, and documentation of occluded coronary artery with coronary angiography. The samples were collected from STEMI patients (n = 80) by venipuncture at three time points. The time points include the time of presentation at cardiac catheterization laboratory, but before reperfusion (pre-PCI), 2 h post successful reperfusion (2 h post-PCI), and 24 h post successful reperfusion (24 h post-PCI). To better understand the late response to reperfusion, in a sub-section of the STEMI cohort (n = 30), blood samples were prospectively collected at two additional time points, including 48 h after reperfusion (48 h post-PCI) and 30 days post successful reperfusion. For the age/sex-matched comparison population (n = 50), blood from patients referred to for diagnostic coronary angiography without any evidence of coronary disease was collected after angiography. Plasma was collected after centrifugation (2,500g, 5 min, 4 °C) and was immediately stored at - 80 °C until required. The average time between blood collection to plasma separation and aliquoting was less than 20 min. The study was approved by St. Boniface General Hospital and the University of Manitoba Research Ethics Board. Written informed consent was obtained from all study participants, and the study was conducted in compliance with the principles of the Declaration of Helsinki.

### Method details

#### Lipid standards and solvents

Tetrahydrofuran, methanol (Optima LC/MS grade), and water were purchased from Fisher Chemical (Mississauga, ON). Ammonium formate and 1-butanol (analytical grade) were purchased from Sigma-Aldrich (St Louis, MO, USA). Chloroform (analytical grade) was purchased from Millipore Sigma (Oakville, ON). Lipid internal standards (ISTD) for dihydroceramide (dhCer 8:0), ceramide (Cer 17:0), sphingomyelin (SM 12:0), lysophosphatidylcholine (LPC 13:0), phosphatidylcholine (PC 13:0_13:0), oxidised phospholipids (PC 9:0_9:0), lysophosphatidylethanolamine (LPE 14:0), phosphatidylethanolamine (PE 17:0_17:0), phosphatidylglycerol (PG 17:0_17:0), and phosphatidylserine (PS 17:0_17:0) were purchased from Avanti Polar Lipids (Alabaster, AL, USA). Acylcarnitine (Acylcarnitine 3:0 (d5), Acylcarnitine 14:0 (d3)), cholesteryl ester (CE 18:0 (d6)), and fatty acid (FA 15:0 (d3)) were purchased from CDN Isotopes (Pointe-Claire, QC). Diacylglycerol (DG 15:0_15:0) was purchased from LGC Standards (Manchester, NH, USA). Monohexosylceramide (HexCer 16:0 (d3)), dihexosylceramide (Hex2Cer 16:0 (d3)), and trihexosylceramide (Hex3Cer 17:0) were purchased from MJS BioLynx (Brockville, ON). Triacylglycerol (TG 17:0_17:0_17:0) was purchased from Sigma-Aldrich (St Louis, MO, USA). Synthetic standards for phosphatidylcholines (PC) containing oxidized phospholipids (OxPL) such as 1-palmitoyl-2- (5-oxovaleroyl)-*sn*-glycero-3-phosphocholine (POVPC), 1-palmitoyl-2-(9-oxo)nonanoyl-*sn*-glycero-3-phosphocholine (PONPC), 1-palmitoyl-2-glutaroyl-*sn*-glycero-3-phosphocholine (PGPC), and 1-Palmitoyl-2-azelaoyl-*sn*-glycero-3-phosphocholine (PAzPC) were obtained from Avanti Polar Lipids. 1-(palmitoyl)-2-(5-keto-6-octene-dioyl)-3-phosphocholine (KOdiAPC), and 1-palmitoyl-2-(4-keto-dodec-3-ene-dioyl)-*sn*-glycero-3-phosphocholine (KDdiAPC) were purchased from Cayman Chemicals (Ann Arbor, MI, USA). Cholesteryl ester (CE) and fatty acid (FA) synthetic standards were purchased from Nu-Chek-Prep (Elysian, MN, USA).

#### Lipid extraction

Lipid extraction was done using chloroform and methanol (Weir et al., 2013; Meikle et al., 2011). Briefly, plasma samples were thawed, and 10 μL of plasma was dispensed to a 1.5 mL polypropylene tube (Eppendorf). To the tube, 30 μL of lipid internal standards (ISTD) in chloroform/methanol (1:1, v/v) was added alongside 200 μL of chloroform/methanol (2:1, v/v). Table S1 contains the complete list of lipid abbreviations. ISTDs were either odd-chain or deuterated and are not present endogenously (Table S2). The mixture was vortexed on a rotary mixer for 10 min and sonicated in a water bath at room temperature (RT) for 30 min. The mixture was subsequently allowed to settle for 20 min and then centrifuged (20,000g, 20 min, RT). The upper lipid-containing phase was transferred into a clean polypropylene tube and dried under a stream of nitrogen gas at room temperature. Lipids were reconstituted in 50 μL water-saturated 1-butanol and sonicated for 10 min. Finally, 50 μL of 10 mM ammonium formate in methanol was added to the final lipid extract. The extract was centrifuged (10,000g, 10 min, RT), and 80 μL of the supernatant was transferred into the micro insert in sample vials for lipid analysis.

#### Lipid separation

Lipids were separated on a reverse-phase liquid chromatography-electrospray ionization tandem mass spectrometry (LC/ESI-MS/MS) platform using a Prominence chromatographic system (Shimadzu Corporation, OR, USA). Instrument control and data processing were done with Analyst v1.6 and Multi-Quant v2.1 software (AB Sciex, MA, USA). The separation was achieved on a Zorbax C18, 1.8 μm, 50 × 2.1 mm column (Agilent Technologies, Mississauga, ON). The flow rate was set to 300 μL/min using a linear gradient of mobile phase A and mobile phase B. Mobile phase A and B consisted of tetrahydrofuran, methanol, and water in the ratio 20:20:60 (v/v/v) and 75:20:5 (v/v/v), respectively. Both A and B contained 10 mM ammonium formate. The elution program was as follows: start with 0% solvent B; increase to 100% B at 8.00 min; maintained at 100% B for 2.5 min; and back to 0% B over 0.5 min. The column was finally re-equilibrated to the starting conditions (0% mobile phase B) for 3 min before the next sample injection. Diacylglycerol (DG) and triacylglycerol (TG) species were separated in an isocratic fashion (100 μL/min) by employing 85% mobile phase B over 8 min. The analytical column and autosampler were maintained at 50°C and 25°C, respectively, during the analysis. The injection volume was 5 μL.

#### Mass spectral analysis

Lipids eluted from the HPLC system were introduced into the AbSciex 4000 QTRAP triple quadrupole linear hybrid mass spectrometer. The mass spectrometer was operated in scheduled Multiple Reaction Monitoring (MRM) mode. In total, 322 unique lipids spanning 25 different lipid classes/subclasses were screened for targeted semi-quantitation (Table S3). All lipid species other than fatty acids were scanned in positive electrospray ionization mode [ESI+] mode. The individual lipids in each lipid class were identified by lipid class-specific precursor ions or neutral losses (Table S1). Lipids were then quantified by comparing the deisotoped lipid peak areas against those of the class-specific ISTDs added before lipid extraction. The linearity plot of lipid internal standards (ISTDs) in plasma is shown in Figures S1–S3. Lipids were represented by the total carbon number of the fatty acids. The collision energy and declustering potential for each lipid class were fixed individually using flow injection analysis (Table S3). In the ESI+ mode, the instrument settings were optimised as follows: curtain gas (psi), 26; collision gas (nitrogen), medium; ion spray voltage (V), 5500; temperature (°C), 500.0; ion source gas 1 (psi), 40.0; ion and source gas 2 (psi), 30.0. The MRM detection window was fixed between 45 and 90s depending upon the chromatographic peak width of the lipid class. Isobaric species within the same class, such as PC(O) and PC(P), exhibited clear separation in this method (Figure S4A). Also, molecular species within the same lipid class, which differ only in the number of double bonds, were well separated chromatographically (Figures S4B and S4C). MRM transitions were established to detect phosphocholine (PC) containing oxidized phospholipids (OxPL) molecules in ESI + mode using a product ion of 184.3 *m*/*z*, matching the cleaved phosphocholine moiety. Six commercially available OxPL standards (PONPC, POVPC, PGPC, PAzPC, KOdiAPC, and KDdiAPC) were injected, and accurate peak assignments were made based upon retention times and mass transitions (Figure S4D). Additionally, M+1/M+2 isotopes were scanned for high-intensity lipid species to prevent detection saturation. The detection of neutral lipids, including CE, DG, and TG species in ESI + mode, was reported using ammonium salts generated in the presence of ammonium formate contained in the mobile phase.

##### Correction factors

Generally, lipid species of the same class have the same response factors. Certain lipid classes, including DG, TG, and CE, present with a rather complicated mass spectrum due to the fragmentation of ammoniated adducts, leading to the loss of fatty acid(s) and ammonia (Weir et al., 2013). Due to the differential fragmentation efficiency of different acyl chain lengths, there is a considerable variation in response factors between species in these classes. Consequently, additional correction factors (CF) were applied for these lipid species (Table S3). In DG species, if both fatty acyl chains in DG species were different, we applied a correction factor (CF) of 0.5. For TG species, if all the three fatty acyl chains were different, we applied a correction factor of 0.33. If two of the three acyl groups were the same in TG, we applied a correction factor of 0.66. To calculate the instrument response for CE, we prepared an equimolar mixture of seven commercially available CE species comprising three compounds varying in chain length (CE 16:0, CE 20:0, and CE 20:0), two monounsaturated species (CE 16:1 and CE 18:1), and two polyunsaturated species (CE 18:2 and CE 20:4). CE 23:0 was used as an internal standard (100 μM). After extrapolating for carbon chain length and double bonds, we found that saturated CE species were fitted by the following linear equation, y = 0.13x-0.71, where ‘y’ is the response factor, and ‘x’ is the carbon chain length. Monounsaturated species were characterized by the linear equation y = 1.13x-0.23 and polyunsaturated species by y = −0.07x+1.54. Other lipid species were not corrected.

##### Fatty acids

Acidic compounds, namely free fatty acids (FA), were detected in the negative ESI mode (ESI-) using a “pseudo-molecular” multiple reaction monitoring (MRM) as previously described (Hellmuth et al., 2012). In the ESI- mode, ion source parameters including collision energy and declustering potential were optimized by flow injection analysis using commercially bought FA standards. Accordingly, the turbo ion spray voltage was set to −4500 V and the source temperature to 500°C. Nitrogen was used as collision gas, and its pressure was set to “medium”. The nebulizer gas was set to 40 psi, the auxiliary gas (GS2) to 30 psi, and the curtain gas to 26 psi. Thirteen commercially available FA standards were purchased, and accurate peak assignments were made based upon retention times and mass transitions. Two MRM transitions corresponding to the monoisotopic [M−H]^-^ mass of the FA analyte were monitored for each FA. Though these MRM transitions have the same fragment and product ions, they differ in their collision cell parameters (Figure S5). The first MRM transition with a low collision energy was used for quantification, while the second MRM transition with increased collision energy provided an unambiguous FA identification. In short, the second MRM for each FA served as a second qualification parameter.

#### Quality control

The study followed the guidelines outlined by the lipidomics consortium for analytical quality assurance (Triebl et al., 2020; Burla et al., 2018a). The plasma samples were randomized before analysis. The sample run order was given in Table S4. Six injections of blank extracts, in the beginning, were used to identify the common contaminations. This study used two types of quality control (QC) samples to ensure analytical reproducibility and quality. A plasma quality control (PQC) sample was prepared by pooling equal aliquots (20 μL) from all samples. To assure the stability of the analytical platform, ten PQC samples were run at the beginning of each batch. In addition, PQC samples were intermittently injected throughout each batch to monitor and compensate for inter-batch analytical variability. Also, lipid extracts without matrix (plasma), referred to to as technical quality control (TQC) samples, were analyzed at even intervals during the analytical batch. They were used to monitor the technical variation in LC/MS over time. Additionally, four replicate injections of a global shared reference sample (NIST SRM 1950 plasma (Triebl et al., 2020)) were included in each analytical batch to enable future comparison with different datasets.

#### Data pre-processing and batch alignment

The samples were run in multiple analytical batches, and each batch contained 60 study samples. There were twenty PQC samples within an analytical batch, six TQC samples, four SRM samples, and twelve blank extracts. The mass spectrometer’|'s source was cleaned after two successive batches. To compensate for time-dependent drifts and batch variations, we used a PQC based normalization method known as systematic error removal using random forest (SERRF) as previously described (Fan et al., 2019). The ratio of study samples to PQC samples was kept at 10:1. After batch correction, lipids were considered reproducible only if their coefficient of variation (CoV) among PQC samples was <20% and their highest mean was not in the blank extracts. Outliers with CoV <20% were removed (n = 31), and only 291 lipid species were found eligible for subsequent downstream analysis. Principal component analysis (PCA) was used to evaluate the data consistency for all samples across all batches (Figure S6). The tight clustering of identical samples (PQC, SRM) in the PCA plot validates the data quality after SERRF normalization.

### Quantification and statistical analysis

Statistical analysis was done using the SPSS v24 (IBM Corporation, Armonk, NY, USA) software. Baseline participants' characteristics were presented as mean ± SD for continuous variables with normal distribution, median (25th, 75th percentiles) for continuous variables with nonnormal distribution, and count (percentage) for categorical variables. The normality assumption of data was checked using the Shapiro-Wilk test. Based on the data distribution, the Chi-square test was used to assess differences in data across groups for categorical variables, and the Mann-Whitney U test or Student’|'s t-test was used for continuous variables as applicable. All tests were 2-sided, and p values < 0.05 were considered statistically significant.

The lipid data were log-transformed before statistical analysis. A repeated measures ANOVA with a Greenhouse-Geisser correction was used to find the differentially regulated lipid species across time points. For pairwise comparisons, only corrected p < 0.05 were considered statistically significant. The “corrected p” denotes the false discovery rate (FDR) correction using the Bonferroni technique. The built-in ‘pheatmap’ package in R statistical software v3.5.2 (https://www.r-project.org) was used to compute and draw the clustered heatmap. The general linear regression model in SPSS employing the ‘Enter’ method was used to examine the associations between delta troponin (Δ cTnT) and lipid species, adjusting for the other covariates.

## Data Availability

The lipidomics data have been deposited to the publicly available EMBL-EBI MetaboLights database with the identifier MTBLS3839. This paper does not report original code. Any additional information required to reanalyze the data reported in this paper is available from the lead contact upon request.

## Supplemental information

Document S1. Figures S1–S6, Tables S1, S2, S4–S8 and S12–S17

Table S3. LC/MS conditions for lipid identification in human plasma (Related to STAR methods)

Table S9. Plasma individual lipids differentially regulated in 2 h post-PCI (t1) vs. pre-PCI (t0) (Related to Figure 2A)

Table S10. Plasma individual lipids differentially regulated in 24 h post-PCI (t2) vs. 2 h post-PCI (t1) (Related to Figure 2B)

Table S11. Plasma individual lipids differentially regulated in 24 h post-PCI (t2) vs. pre-PCI (t0) (Related to Figure 2C)
