# Supplementary material for: Impact of myocardial reperfusion on human plasma lipidome
Source: iScience. 2022 Jan 29;25(2):103828. doi: 10.1016/j.isci.2022.103828 (PMC8850755; doi:10.1016/j.isci.2022.103828)
Supplement: Document S1. Figures S1–S6, Tables S1, S2, S4–S8 and S12–S17 [file mmc1.pdf]

iScience, Volume *25*

Supplemental information

Impact of myocardial reperfusion
on human plasma lipidome

Arun Surendran, Negar Atefi, Umar Ismail, Ashish Shah, and Amir Ravandi

Figure S1: **Linearity plot of lipid internal standards (Cer 17:0, dhCer 8:0, PC 13:0_13:0, PE 17:0_17:0, LPC 13:0, LPE 14:0, PS 17:0_17:0 and PG 17:0_17:0) in plasma** (related to STAR methods)

Serial dilutions of lipid internal standards (ISTDs) in a plasma quality control (PQC) sample were used to generate the response curves. **Table S1** contains the complete list of lipid abbreviations.

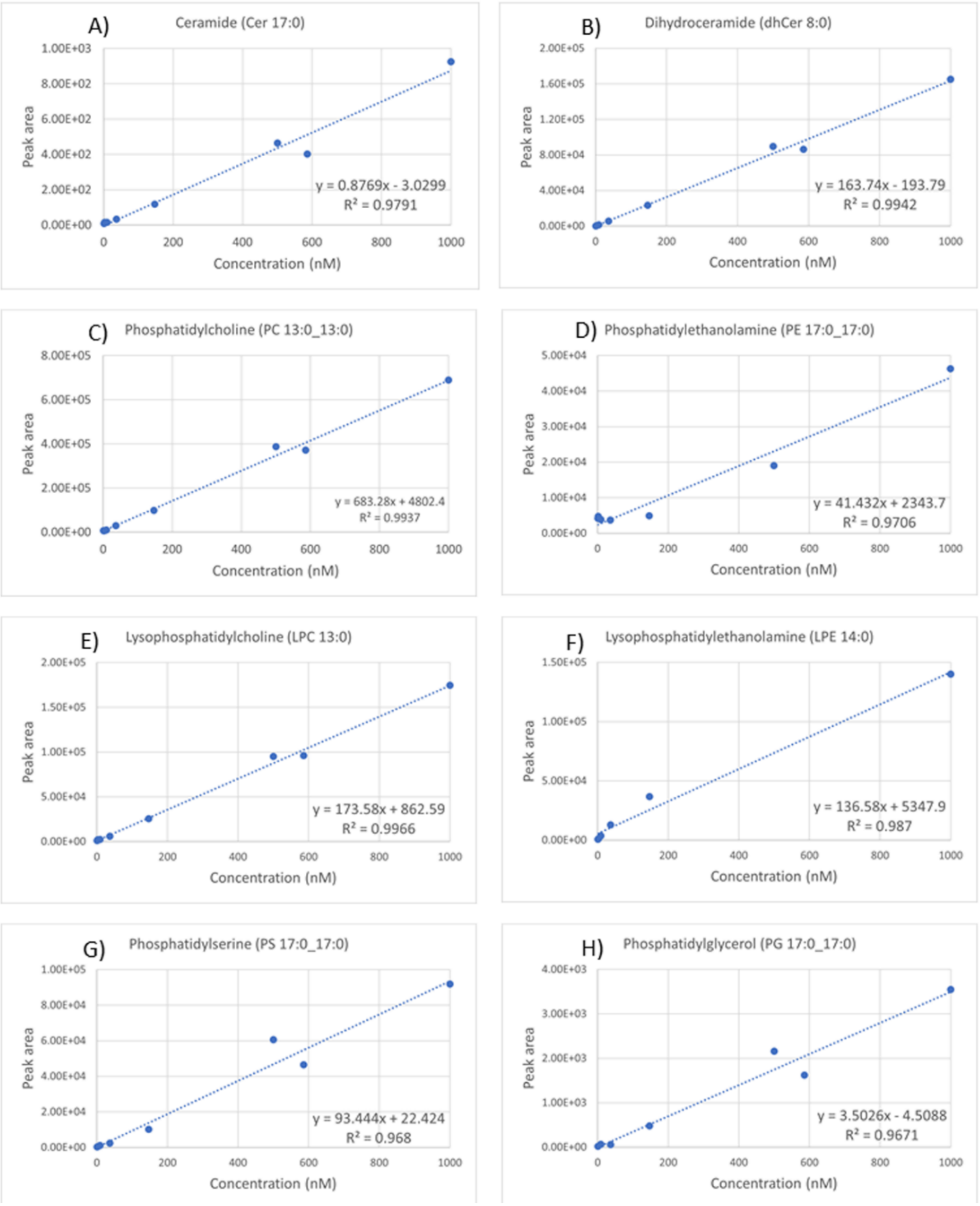

Figure S2: Linearity plot of lipid internal standards (SM 12:0, HexCer 16:0 (d3), Hex2Cer 16:0 (d3), Hex3Cer 17:0, Acylcarnitine 3:0 (d5), Acylcarnitine 14:0 (d3), Diacylglycerol (DG 15:0_15:0) and Triacylglycerol (TG 17:0_17:0_17:0)) in plasma (related to STAR methods)

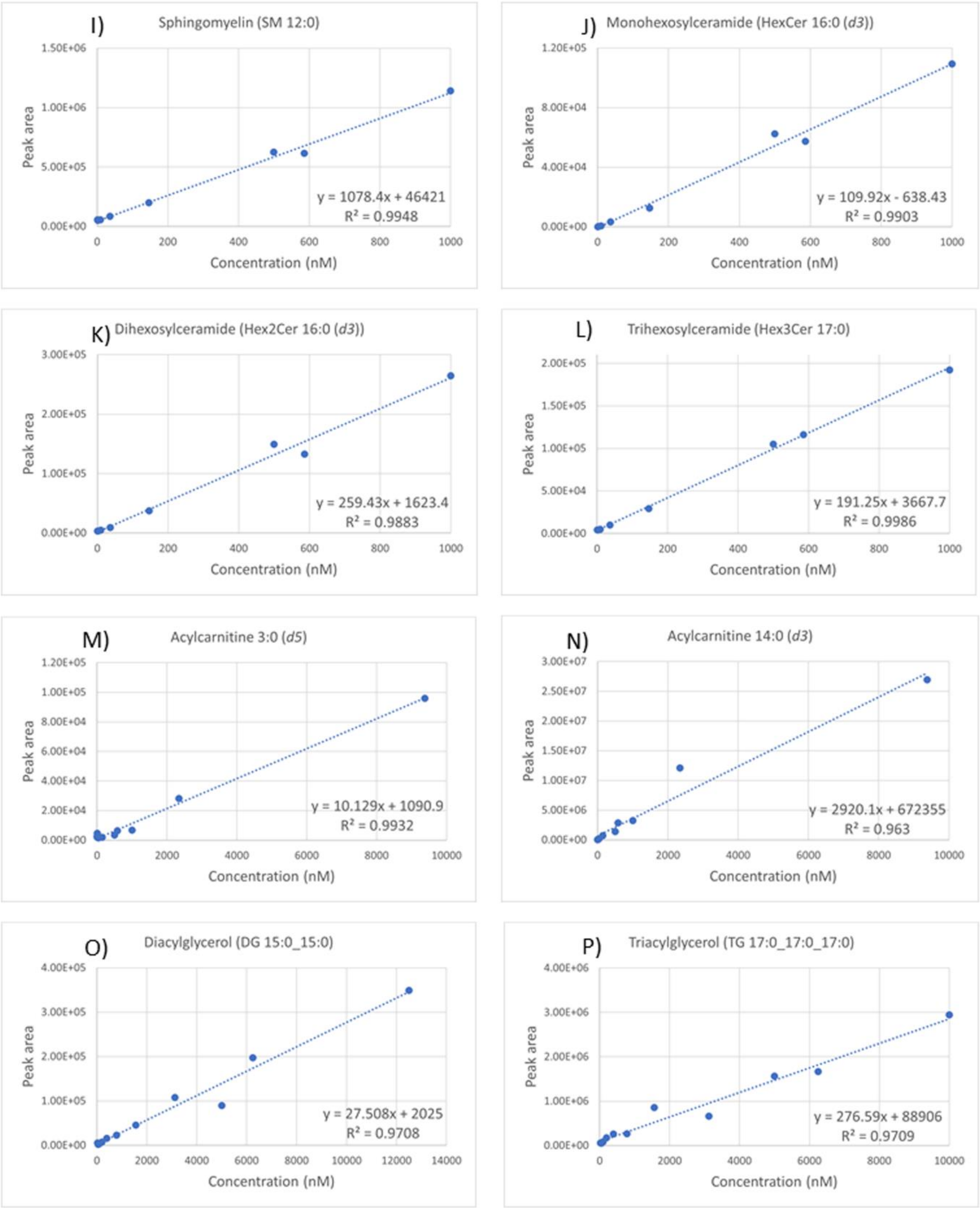

Figure S3: Linearity plot of cholesteryl ester (CE 18:0 (d6)), and fatty acid (FA 15:0 (d3)) in plasma (related to STAR methods)

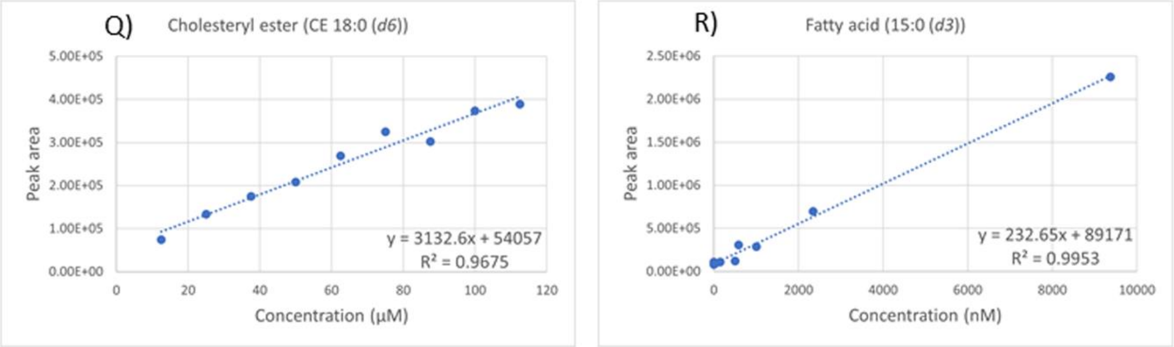

Figure S4: Chromatographic separation of lipid species in ESI+ mode (related to STAR methods): Figure (A) shows separation of two isobaric species of PC in plasma scanned using the same transition 718.5/184.3, (B) shows separation of two acylcarnitine species in plasma differing in the number of carbon double bonds, (C) shows separation of two LPC species in plasma differing in the number of carbon double bonds and (D) shows separation of an equimolar mixture (100 µM) of six commercially bought oxidized phospholipids (OxPL) standards. **Supplementary Table-1** contains the complete list of lipid abbreviations.

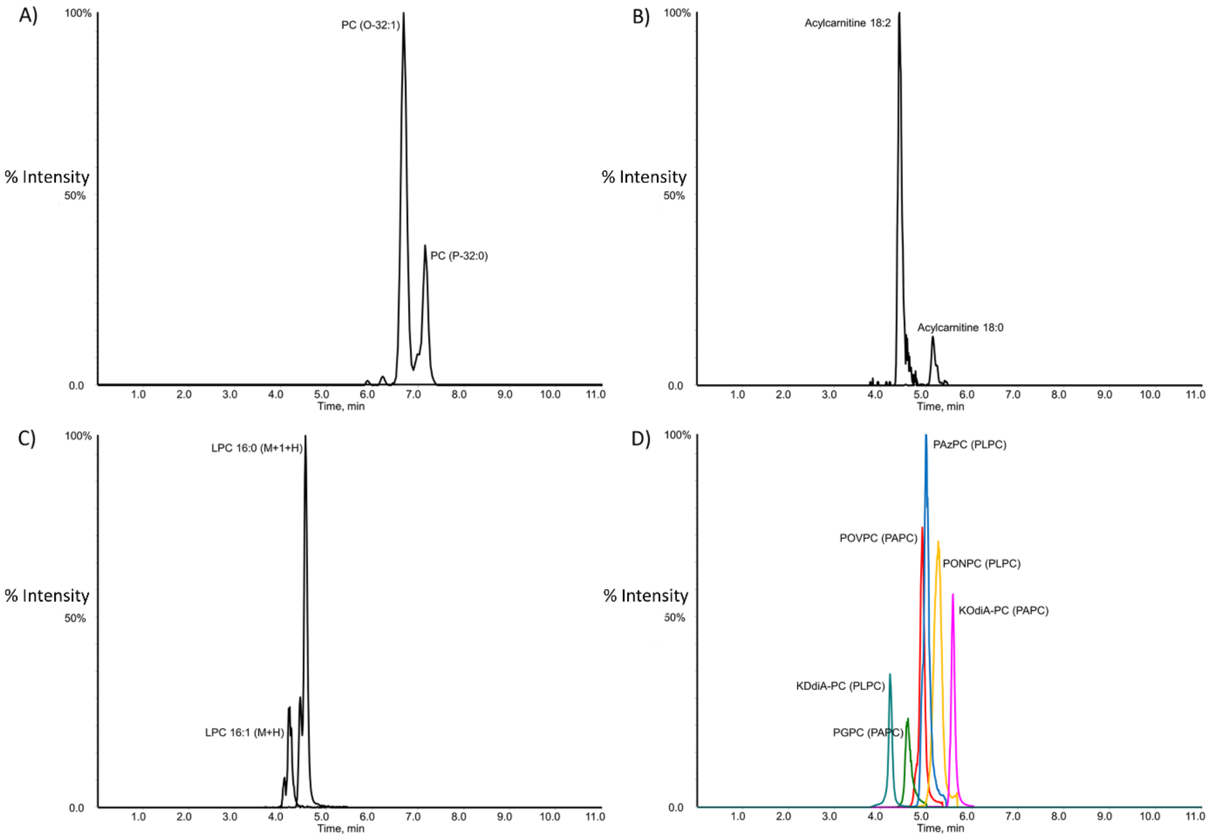

Figure S5: Fatty acids in ESI- mode (related to STAR methods): The figure shows separation of c18 fatty acids in human plasma in ESI- mode. The quantifier ions with low collision energy are shown in blue, while the "pseudo-qualifier" ions with high collision energy are red.

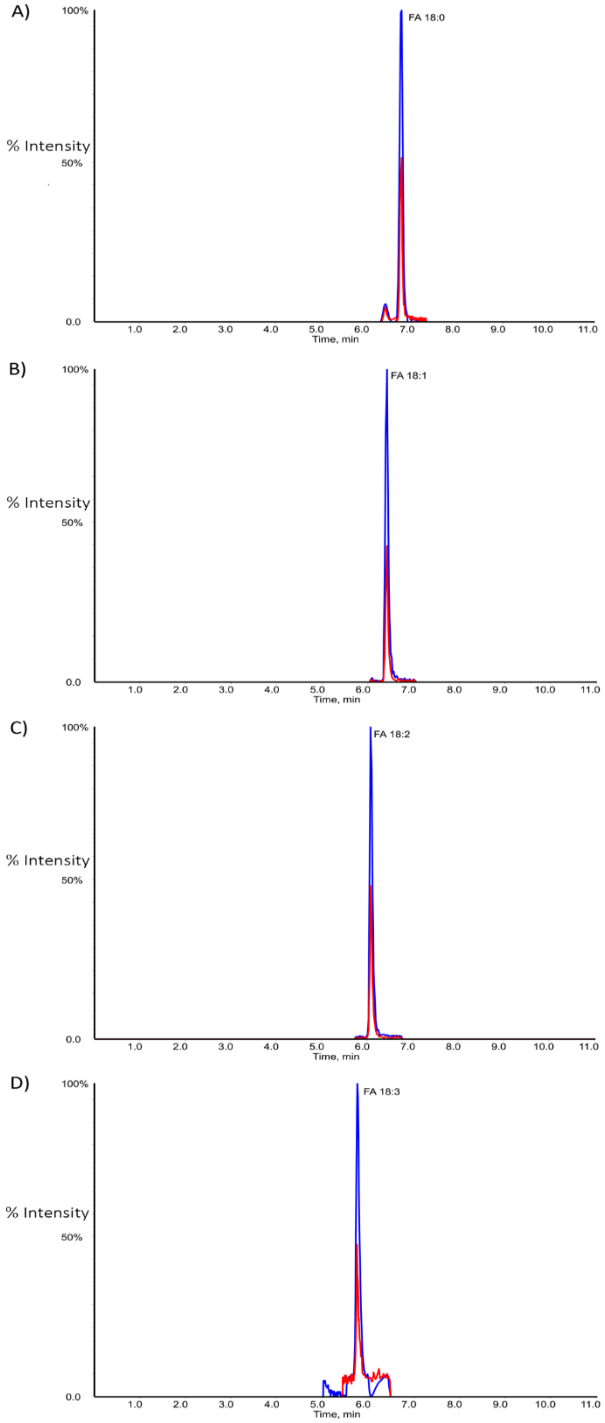

Figure S6: Principal Component Analysis (PCA) plot (related to STAR methods): The tight clustering of the plasma quality control samples (PQC) and global shared reference samples (SRM) shows the data quality after SERRF normalization.

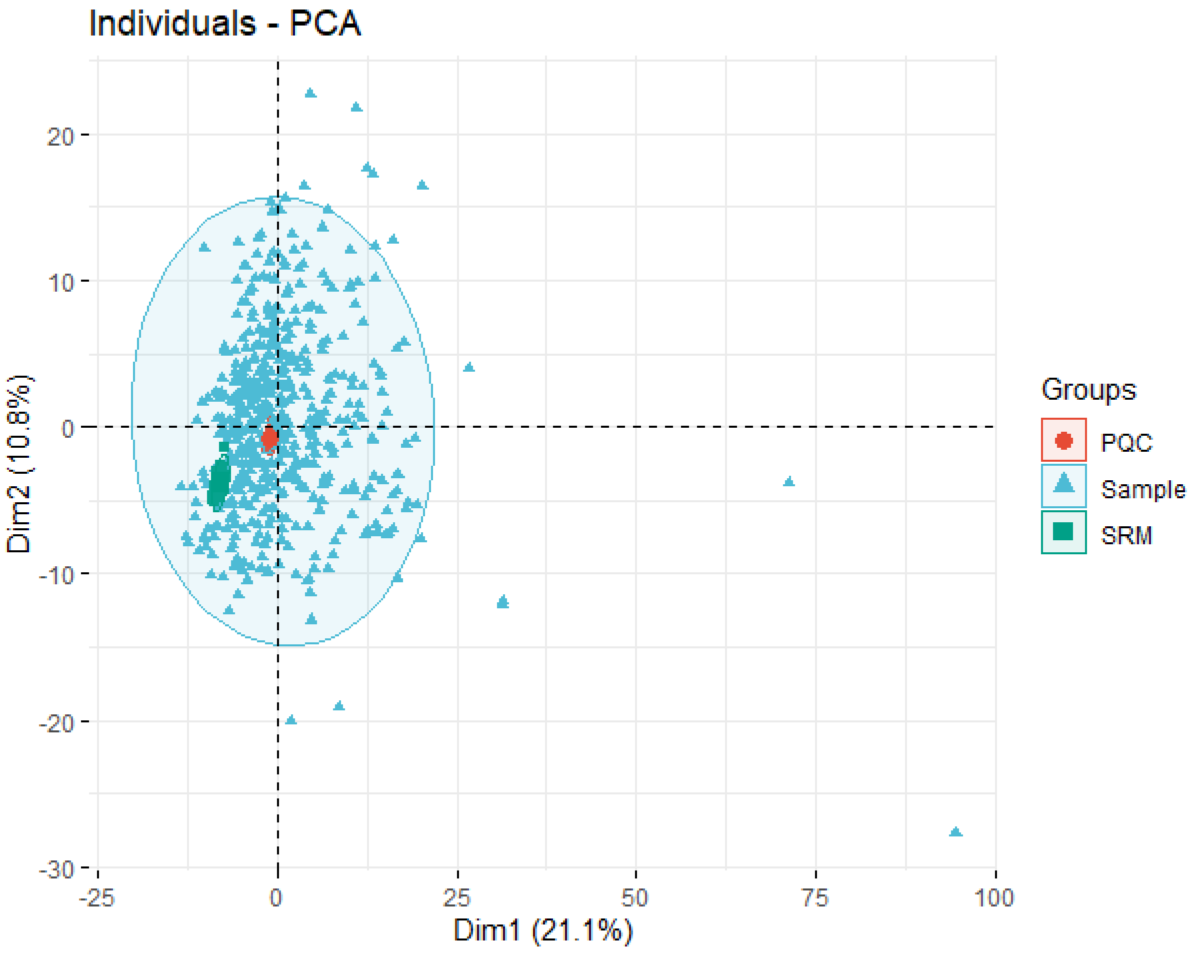

Table S1: Abbreviations for lipids (related to STAR methods)

**Class**	**Abbreviation**
Dihydroceramide	dhCer
Ceramide	Cer
Monohexosylceramide	HexCer
Dihexosylceramide	Hex2Cer
Trihexosylcermide	Hex3Cer
GM3 ganglioside	GM3
Sphingomyelin	SM
Phosphatidylcholine	PC
Oxidized phospholipids	OxPL
Alkylphosphatidylcholine	PC(O)
Phosphatidylcholine plasmalogen	PC(P)
Lysophosphatidylcholine	LPC
Lysoalkylphosphatidylcholine	LPC(O)
Phosphatidylethanolamine	PE
Alkylphosphatidylethanolamine	PE(O)
Phosphatidylethanolamine plasmalogen	PE(P)
Lysophosphatidylethanolamine	LPE
Phosphatidylinositol	PI
Phosphatidylserine	PS
Phosphatidylglycerol	PG
Cholesterol ester	CE
Diacylglycerol	DG
Triacylglycerol	TG
Fatty acids	FA
1-palmitoyl-2- (5-oxovaleroyl)-sn-glycero-3-phosphocholine	POVPC
1-palmitoyl-2-(9-oxo)nonanoyl-sn-glycero-3-phosphocholine	PONPC
1-palmitoyl-2-glutaroyl-sn-glycero-3-phosphocholine	PGPC
1-Palmitoyl-2-azelaoyl-sn-glycero-3-phosphocholine	PAzPC
1-(palmitoyl)-2-(5-keto-6-octene-dioyl)-3-phosphocholine	KOdiAPC
1- palmitoyl-2-(4-keto-dodec-3-ene-dioyl)-sn-glycero-3-phosphocholine	KDdiAPC

Table S2: Experimental conditions for lipid identification and quantification (related to STAR methods)

Lipid class	Lipid species	Internal Standard	Parent Ion	µM/ 30 µl	Fragmentation
dhCer	6	dhCer 8:0	[M+H]+	0.5	PIS, m/z 284.4
Cer	6	Cer 17:0	[M+H]+	5	PIS, *m/z* 264.4
HexCer	6	MHC 16:0 (*d3*)	[M+H]+	0.5	PIS, *m/z* 264.4
Hex2Cer	6	DHC 16:0 (*d3*)	[M+H]+	0.5	PIS, *m/z* 264.4
Hex3Cer	6	THC 17:0	[M+H]+	0.5	PIS, *m/z* 264.4
GM3	6	THC 17:0	[M+H]+	0.5	PIS, *m/z* 264.4
SM	20	SM 12:0	[M+H]+	0.5	PIS, *m/z* 184.3
PC	31	PC 13:0_13:0	[M+H]+	0.5	PIS, *m/z* 184.3
OxPL	6	PC 9:0_9:0	[M+H]+	0.5	PIS, *m/z* 184.3
PC(O)	14	PC 13:0_13:0	[M+H]+	0.5	PIS, *m/z* 184.3
PC(P)	6	PC 13:0_13:0	[M+H]+	0.5	PIS, *m/z* 184.3
LPC	19	LPC 13:0	[M+H]+	0.5	PIS, *m/z* 184.3
LPC(O)	6	LPC 13:0	[M+H]+	0.5	PIS, *m/z* 104.1
PE	18	PE 17:0_17:0	[M+H]+	0.5	NL, 141 Da
PE(O)	12	PE 17:0_17:0	[M+H]+	0.5	NL, 141 Da
PE(P)	7	PE 17:0_17:0	[M+H]+	0.5	NL, 141 Da
LPE	7	LPE 14:0	[M+H]+	0.5	NL, 141 Da
PI	17	PE 17:0_17:0	[M+NH4]+	0.5	NL, 277 Da
PS	7	PS 17:0_17:0	[M+H]+	0.5	NL, 185 Da
PG	4	PG 17:0_17:0	[M+NH4]+	0.5	NL, 189 Da
CE	20	CE 18:0 (*d6*)	[M+NH4]+	20	PIS, *m/z* 369.3
Acylcarnitine (<C14)	11	Acylcarnitine 3:0 (*d5*)	[M+H]+	5	PIS, *m/z* 85.0
Acylcarnitine (C>14)	5	Acylcarnitine 14:0 (*d3*)	[M+H]+	0.1	PIS, *m/z* 85.0
DG	20	DG 15:0_15:0	[M+NH4]+	5	NL, fatty acid
TG	43	TG 17:0_17:0_17:0	[M+NH4]+	5	NL, fatty acid
FA	13	FA 15:0 (*d3*)	[M-H]-	0.5	Pseudo MRM

Abbreviations: PIS, precursor ion scan; NL, neutral loss scan; MRM, Multiple reaction monitoring. **Table S1** contains the complete list of lipid abbreviations.

Table S4: Sample run order (related to STAR methods)

Injection No	Sample type	
1	Extracted blank-1	Six blank injections in the beginning of each batch.

5	Extracted blank-6	
6	System conditioning QC-1	Ten PQC in the beginning of each batch for system equilibration.

15	System conditioning QC-10	
16	PQC-1	Injecting various QC samples (PQC, TQC and SRM) to monitor system stability and technical variation.
17	PQC-2	
18	TQC-1	
19	TQC-2	
20	SRM-1	
21	SRM-2	
22	PQC-3	
23	Study sample-1	Study sample injection starts
24	Study sample-2	

32	Study sample-10	
33	Extracted blank-6	Blank injection after 10 consecutive study samples injection.
34	PQC-4	
35	Study sample-11	

44	Study sample-20	
45	Extracted blank-7	
46	PQC-5	PQC injection after 20 study samples injection.
47	TQC-3	TQC injection after 20 study samples injection.

Abbreviations: PQC, plasma quality control; TQC, technical quality control; SRM, NIST SRM 1950 plasma

Table S5: Lipids shared or unique between different time points.
(Related to Figure 1C)
A:92 common lipids in "*t1 vs. t0*", "*t2 vs. t1*" and "*t2 vs. t0*"
B:15 lipids exclusive to "*t1 vs. t0*"
C:20 lipids exclusive to "*t2 vs. t1*"
D:6 lipids exclusive to "*t2 vs. t0*"

No	A	B	C	D
1	Acylcarnitine 2:0	DG 18:0_20:4	CE 18:2	Acylcarnitine 16:0
2	CE 22:6	DG 18:1_20:3	CE 22:4	Acylcarnitine 20:0
3	Cer 18:0	DG 18:1_20:4	Cer 24:0	CE 22:5
4	Cer 22:0	LPC(O-24:1)	GM3 16:0	PC(O-36:3)
5	DG 16:0_18:1	LPE 16:0	LPC (O-24:0)	PGPC
6	DG 16:0_18:2	LPE 18:0	PC 29:0	PONPC
7	DG 16:0_22:5	PC 34:3	PC 33:1	
8	DG 16:1_18:1	PC 35:4	PC 36:4	
9	DG 18:0_18:1	PC 36:3	PC 36:6	
10	DG 18:0_18:2	PC(O-36:4)	PC 37:5	
11	DG 18:1_18:1	PE 35:2	PC 37:6	
12	DG 18:1_18:2	PE(O-36:2)	PC 38:3	
13	DG 18:1_18:3	PE(O-40:6)	PC(O-34:1)	
14	DG 18:2_18:2	PE(P-40:5)	PC(O-36:1)	
15	LPC 14:0	SM 39:1	PC(O-38:5)	
16	LPC 16:1		PC(O-40:7)	
17	LPC 18:1		PE 36:3	
18	LPC 20:0		SM 38:1	
19	LPC 20:1		SM 41:2	
20	PC 34:1		THC 16:0	
21	PC 38:4			
22	PC 38:5			
23	PC(O-32:2)			
24	PE 34:1			
25	PE 34:2			
26	PE 36:1			
27	PE 36:2			
28	PE(O-34:2)			
29	PE(O-36:3)			
30	PE(O-36:4)			
31	PE(O-36:5)			
32	PE(O-38:5)			
33	PE(O-38:6)			
34	PE(P-34:2)			
35	PE(P-38:4)			
36	PG 34:1			

37	PG 36:1
38	PG 36:2
39	PI 32:0
40	PI 32:1
41	PI 34:0
42	PI 34:1
43	PI 36:0
44	PI 36:1
45	PI 36:2
46	PI 36:3
47	PI 36:4
48	PI 38:2
49	PI 38:5
50	PI 38:6
51	PI 40:4
52	PI 40:6
53	SM 36:1
54	SM 36:2
55	SM 36:3
56	TG 48:2
57	TG 48:2
58	TG 48:2
59	TG 48:2
60	TG 48:3
61	TG 48:3
62	TG 49:1
63	TG 49:1
64	TG 49:1
65	TG 50:1
66	TG 50:1
67	TG 50:2
68	TG 50:2
69	TG 50:3
70	TG 50:3
71	TG 50:3
72	TG 50:4
73	TG 51:0
74	TG 51:1
75	TG 51:2
76	TG 51:2
77	TG 51:2
78	TG 52:1
79	TG 52:2
80	TG 52:3

81	TG 52:3
82	TG 52:4
83	TG 52:4
84	TG 53:2
85	TG 54:2
86	TG 54:3
87	TG 54:4
88	TG 54:4
89	TG 54:5
90	TG 54:6
91	TG 56:6
92	TG 58:8

Table S6. Plasma lipid classes differentially regulated in 2 h post-PCI (*t1*) vs. pre-PCI (*t0*) (Related to Figure 1D).

No	Lipid Class	Percentage difference, *t1 vs. t0* (%)	SEM	p-value	p-value (Bonferroni)*
1	Cer	-15.2324	2.310853	1.64E-08	1.95E-08
2	dhCer	-4.43055	4.005675	0.456165	0.788089
3	HexCer	-7.98452	2.738544	0.001249	0.014076
4	Hex2Cer	1.068409	3.74162	0.226997	1
5	Hex3Cer	-6.62879	3.257072	0.028895	0.162756
6	GM3	-8.99302	3.355359	0.019449	0.028177
7	SM	-8.13677	1.602941	1.51E-08	7.79E-06
8	PC	-2.6724	1.950236	0.003502	0.537792
9	PC(O)	-4.16148	2.050878	0.021878	0.149576
10	PC(P)	-1.55904	2.690074	0.02455	1
11	LPC	-12.9599	2.777559	1.14E-12	6.61E-05
12	LPC(O)	-8.2038	2.556478	0.001856	0.007021
13	PE	-3.9156	2.05952	3.05E-12	1.81E-01
14	PE(O)	-10.895	1.889008	4.11E-09	4.67E-07
15	PE(P)	-8.86941	1.827694	8.52E-11	1.96E-05
16	LPE	-4.50767	3.689748	5.53E-20	6.62E-01
17	PI	-15.7131	2.402064	1.89E-13	2.30E-08
18	PS	8.600936	7.631153	0.573445	1
19	PG	-17.0643	4.698144	1.44E-12	1.89E-03
20	CE	-11.4486	3.850719	0.000361	0.011194
21	Acylcarnitine	-15.0511	5.075217	1.58E-08	0.013253
22	**OxPL**	**29.87915**	10.40869	0.001854	0.026933
23	**DG**	**-29.9244**	4.591791	4.35E-20	5.21E-08
24	**TG**	**-24.2858**	4.048233	1.74E-18	2.91E-07
25	FA	-12.6519	5.572163	6.35E-25	1.11E-01

p-value: Repeated measures ANOVA with a Greenhouse-Geisser correction.
*: Corrected p-value after adjustment for multiple comparisons using Bonferroni. Corrected p<0.05 are highlighted in light red.
Abbreviation: SEM, Standard Error Mean

Table S7. Plasma lipid classes differentially regulated in 24 h post-PCI (*t2*) vs. 2 h post-PCI (*t1*) (Related to Figure 1E**).**

No	Lipid Class	Percentage difference, *t2 vs. t1* (%)	SEM	*p*-value	*p*-value (Bonferroni)*
1	Cer	10.74667427	2.553694	1.64E-08	2.12E-04
2	dhCer	4.220715251	4.020922	0.456165	0.869953095
3	HexCer	9.862223428	2.868704	0.001249	0.002968361
4	Hex2Cer	4.803903456	3.390393	0.226997	0.494423818
5	Hex3Cer	7.358663075	2.741552	0.028895	0.027425159
6	GM3	7.406122737	3.380285	0.019449	0.09786463
7	SM	9.409117388	1.522558	1.51E-08	8.17E-08
8	PC	6.676782089	1.817695	0.003502	0.001288246
9	PC(O)	5.195524759	1.470115	0.021878	0.002083447
10	PC(P)	-5.412763068	1.715756	0.02455	0.006849897
11	LPC	-16.50162822	3.909369	1.14E-12	2.50E-04
12	LPC(O)	8.206641183	2.453838	0.001856	0.003996552
13	PE	26.24691618	3.013101	3.05E-12	1.81E-12
14	PE(O)	-8.808699977	2.901976	4.11E-09	1.50E-02
15	PE(P)	-11.56217023	2.596377	8.52E-11	1.18E-04
16	LPE	-37.71380088	4.330246	5.53E-20	2.85E-12
17	PI	-10.86240355	3.105658	1.89E-13	2.33E-03
18	PS	-7.454027394	7.782289	0.573445	0.924246429
19	PG	46.3960175	5.62962	1.44E-12	1.10E-10
20	CE	18.22884219	4.286609	0.000361	0.000224955
21	Acylcarnitine	-17.70756971	5.250324	1.58E-08	0.00340701
22	OxPL	6.568571315	9.565957	0.001854	1
23	**DG**	**64.06186107**	4.376867	4.35E-20	1.38E-21
24	**TG**	**58.62646089**	4.712544	1.74E-18	3.96E-18
25	FA	-67.16443932	5.154134	6.35E-25	1.45E-19

p-value: A repeated measures ANOVA with a Greenhouse-Geisser correction.
*: Corrected p-value after adjustment for multiple comparisons using Bonferroni. Corrected $p<0.05$ are highlighted in light red.
Abbreviation: SEM, Standard Error Mean

Table S8. Plasma lipid classes differentially regulated in 24 h post-PCI (*t2*) vs. pre-PCI (*t0*) (Related to Figure 1F)**.**

No.	Lipid Class	Percentage difference, *t2 vs. t0* (%)	SEM	*p*-value	*p*-value (Bonferroni)*
1	Cer	-4.526299642	2.478114	1.64E-08	2.12E-01
2	dhCer	-0.34516639	4.135659	0.456165	1
3	HexCer	1.820989799	2.736293	0.001249	1
4	Hex2Cer	5.756988877	3.691018	0.226997	0.348735355
5	Hex3Cer	0.819684699	2.695072	0.028895	1
6	GM3	-1.632415004	3.339083	0.019449	1
7	SM	1.281943359	1.657817	1.51E-08	1.00E+00
8	PC	4.021011798	2.071708	0.003502	0.166790008
9	PC(O)	1.020522411	2.056684	0.021878	1
10	PC(P)	-6.911862204	2.694342	0.02455	0.048811061
11	LPC	-29.51797428	3.225649	1.14E-12	2.48E-13
12	LPC(O)	-0.081773174	2.665744	0.001856	1
13	PE	22.18783761	3.599431	3.05E-12	1.53E-07
14	PE(O)	-19.44120266	2.888806	4.11E-09	1.28E-08
15	PE(P)	-20.23525445	2.855516	8.52E-11	2.28E-09
16	LPE	-42.03891566	4.123581	5.53E-20	4.75E-15
17	PI	-26.2434024	3.251004	1.89E-13	2.86E-11
18	PS	-1.307566634	8.828938	0.573445	1
19	PG	31.24636087	5.577051	1.44E-12	8.11E-06
20	CE	6.588666817	4.799165	0.000361	0.484606778
21	Acylcarnitine	-32.87063766	5.070422	1.58E-08	2.83639E-08
22	OxPL	34.23184598	10.45401	0.001854	0.004596785
23	DG	36.4753469	5.901769	4.35E-20	1.41E-06
24	TG	35.74365838	5.674479	1.74E-18	3.30E-07
25	**FA**	**-74.55166213**	6.240169	6.35E-25	4.49E-17

p-value: A repeated measures ANOVA with a Greenhouse-Geisser correction.
*: Corrected p-value after adjustment for multiple comparisons using Bonferroni. Corrected $p<0.05$ are highlighted in light red.
Abbreviation: SEM, Standard Error Mean

Table S12: Association of lipid species with delta troponin at pre-PCI (*t0*).
(Related to Figure 4A)

		**Unstandardized Coefficients**		**Standardized Coefficients**	**t-value**	***p*-value**	**95.0% CI for B**	
No	Lipids	B	Std. Error	Beta			Lower	Upper
1	**Acylcarnitine 18:2**	**4.87**	**2.23**	**0.28**	**2.182**	**0.033**	**0.41**	**9.32**
2	PI 32:0	-2.85	1.11	-0.33	-2.564	0.013	-5.07	-0.63
3	PE 38:6	-3.80	1.81	-0.28	-2.098	0.040	-7.42	-0.18
4	PI 34:1	-4.15	1.75	-0.30	-2.369	0.021	-7.64	-0.65
5	PI 38:6	-4.35	1.98	-0.31	-2.200	0.031	-8.29	-0.40
6	CE 22:4	-4.49	2.18	-0.26	-2.056	0.044	-8.85	-0.13
7	PE 36:4	-4.65	1.90	-0.29	-2.445	0.017	-8.46	-0.85
8	PI 36:1	-4.68	2.18	-0.27	-2.147	0.035	-9.03	-0.33
9	CE 16:0	-5.95	2.55	-0.28	-2.333	0.023	-11.03	-0.86
10	PC 32:0	-7.61	3.24	-0.29	-2.345	0.022	-14.09	-1.13

Linear regression (delta troponin) - adjusted for age, sex, body mass index (BMI), current smoking, diabetes history, and ischemic time (time from symptom onset to reperfusion)
Positive associations are shown in bold.

Table S13: Association of lipid species with delta troponin at 2 h post-PCI (*t1*).
(Related to Figure 4B)

		**Unstandardized Coefficients**		**Standardized Coefficients**	**t-value**	***p*-value**	**95.0% CI for B**	
No	Lipids	B	Std. Error	Beta			Lower	Upper
1	**SM 38:2**	**9.12**	**4.45**	**0.25**	**2.049**	**0.044**	**0.23**	**18.00**
2	**Acylcarnitine 18:2**	**6.10**	**1.85**	**0.40**	**3.295**	**0.002**	**2.40**	**9.79**
3	**FA 18:2**	**3.81**	**1.87**	**0.26**	**2.044**	**0.045**	**0.09**	**7.54**
4	TG 50:0	-1.79	0.75	-0.34	-2.387	0.020	-3.28	-0.29
5	TG 48:2	-1.94	0.95	-0.28	-2.035	0.046	-3.85	-0.04
6	TG 48:2	-1.97	0.96	-0.28	-2.051	0.044	-3.89	-0.05
7	TG 48:2	-2.03	0.99	-0.29	-2.053	0.044	-4.01	-0.06
8	TG 48:0	-2.04	0.83	-0.34	-2.455	0.017	-3.69	-0.38
9	TG 48:1	-2.17	0.99	-0.32	-2.181	0.033	-4.15	-0.18
10	TG 49:1	-2.18	1.04	-0.30	-2.106	0.039	-4.25	-0.11
11	TG 49:1	-2.21	1.01	-0.31	-2.179	0.033	-4.23	-0.19
12	TG 51:0	-2.34	1.01	-0.33	-2.322	0.023	-4.35	-0.33
13	TG 49:1	-2.43	1.00	-0.35	-2.423	0.018	-4.42	-0.43
14	CE 16:0	-4.54	2.10	-0.26	-2.160	0.034	-8.73	-0.34
15	PE 36:4	-4.97	2.25	-0.27	-2.213	0.030	-9.46	-0.49
16	PE(O-38:5)	-5.00	2.37	-0.26	-2.113	0.038	-9.73	-0.28
17	PE 38:4	-5.06	2.11	-0.29	-2.393	0.020	-9.28	-0.84
18	PE(O-38:4)	-5.25	2.41	-0.26	-2.182	0.033	-10.06	-0.45
19	SM 34:1	-7.72	3.24	-0.28	-2.383	0.020	-14.19	-1.25

Linear regression (delta troponin) - adjusted for age, sex, body mass index (BMI), current smoking, diabetes history, and ischemic time (time from symptom onset to reperfusion)
Positive associations are shown in bold.

Table S14: Association of lipid species with delta troponin at 24 h post-PCI (*t2*). (Related to Figure 4C)

		**Unstandardized Coefficients**		**Standardized Coefficients**	**t-value**	**p-value**	**95.0% CI for B**	
No	Lipids	B	Std. Error	Beta			Lower	Upper
1	PI 34:0	-3.52	1.67	-0.27	-2.107	0.039	-6.86	-0.18
2	LPC 17:1	-4.21	1.99	-0.28	-2.113	0.038	-8.19	-0.23
3	LPC 18:0	-4.65	2.13	-0.27	-2.180	0.033	-8.90	-0.39
4	PC(O-36:4)	-8.02	3.39	-0.29	-2.367	0.021	-14.78	-1.26

Linear regression (delta troponin) - adjusted for age, sex, body mass index (BMI), current smoking, diabetes history, and ischemic time (time from symptom onset to reperfusion)

**Table S15: Clinical demographics for subgroup comparison based on change in troponin concentration ((Δ cTnT)** (Related to Fig.4D)			
	Low Tertile (n=27)	Top Tertile (n=27)	*p* value
Age(years)	63.44 (12.5)	68.14 (12.03)	0.165
Male sex (%)	20 (74.1)	16 (59.3)	0.248
LVEF (%)	58.5 (41.25, 69.25)	50 (40, 64)	0.438
Body mass index (kg/m^2)	26.77 (23.18, 34.71)	26.92(23.40, 28.70)	0.392
**Comorbidity (%)**			
Hypertension	15 (55.6)	12 (44.44)	0.414
Diabetes mellitus	6 (22.2)	2 (7.40)	0.125
Current smoker	10 (37)	7 (25.9)	0.379
Dyslipidemia	18 (66.7)	13 (48.1)	0.169
Hx of CAD	5 (18.5)	3 (11.1)	0.444
**Laboratory data**			
Triglyceride (mmol/l)	1.4 (0.85, 3.15)	1.2 (0.87, 1.42)	0.161
Cholesterol (mmol/l)	4.7 (4.15, 5.6)	4.95 (4.05, 5.70)	0.752
HDL cholesterol (mmol/l)	0.9 (0.7, 1.15)	1.2 (1.05, 1.55)	*0.019*
LDL cholesterol (mmol/l)	2.7 (2.2, 3.5)	3.0 (2.22, 3.55)	0.48
Creatinine (mmol/l)	86 (70, 104)	89 (78, 109)	0.368
**Medications at baseline (%)**			
ASA	7 (25.9)	5 (18.5)	0.513
ACEI/ARB	9 (33.3)	7 (25.9)	0.551
Beta blocker	4 (14.8)	4 (14.8)	1
Statin	9 (33.3)	8 (29.6)	0.77
**Additional parameters**			
Minutes from the onset of chest pain to reperfusion	161 (104, 395)	135 (80, 218)	0.426
Peak CK (Units/L)	331 (237.25, 578.5)	2679 (1803, 3450)	*<0.001*
Peak TnT (ng/L)	762 (499, 1145)	7175 (5740, 10000)	*<0.001*
**Culprit vessel (%)**			
LAD Infarct (%)	13 (48.1)	16 (59.3)	0.413
RCA Infarct (%)	12 (44.4)	8 (29.6)	0.26
Circumflex Infarct (%)	2 (7.4)	3 (11.1)	0.639

Values are reported as mean ± standard deviation (SD), median (25th, 75th percentiles), or count (percentage) as applicable. The Chi-square test was used for categorical variables, while Student's t-test or Mann-Whitney U test was used for continuous variables to assess for statistical significance across sample groups as applicable based on data distribution. Abbreviations: LVEF = left ventricular ejection fraction; Hx of CAD = history of coronary artery disease; HDL = high-density lipoprotein; LDL = low-density lipoprotein; ASA = Acetylsalicylic acid; ACEI = Angiotensin-converting enzyme (ACE) inhibitors; ARB = Angiotensin II receptor blockers;

CK = Creatine kinase; TnT = troponin T; LAD = Left anterior descending coronary artery; RCA = Right coronary artery.

Table S16: **Logistic regression analysis** (Related to Fig.5A-C)
Dependent Variable: Delta troponin (two groups: "low (T1)" and "high (T3)" tertile)

								**95% C.I.for EXP(B)**	
**Sl.No**	**Time**	**Lipid**	**B**	**S.E.**	**Wald**	**p-value**	**Exp(B)**	**Lower**	**Upper**
1	*t0*	PI 34:1	-0.56	0.27	4.38	*0.036*	0.57	0.34	0.96
2	*t1*	AC 18:2	41.58	17.65	5.55	*0.018*	1.15E+18	1087.76	1.20964E+33
3	*t1*	TG 48:1	-1.46	0.60	5.93	*0.015*	0.23	0.07	0.75
4	*t1*	TG 48:2	-3.41	1.46	5.46	*0.019*	0.03	0.00	0.58
5	*t1*	TG 49:1	-6.20	2.69	5.30	*0.021*	0.00	0.00	0.40
6	*t1*	TG 48:2	-14.67	6.84	4.60	*0.032*	0.00	0.00	0.28
7	*t1*	TG 49:1	-12.37	5.32	5.42	*0.020*	0.00	0.00	0.14
8	*t1*	TG 48:0	-0.43	0.18	5.65	*0.017*	0.65	0.46	0.93
9	*t1*	TG 50:0	-1.28	0.58	4.91	*0.027*	0.28	0.09	0.86
10	*t1*	TG 49:1	-5.96	2.51	5.66	*0.017*	0.00	0.00	0.35
11	*t1*	TG 51:0	-47.62	20.64	5.32	*0.021*	0.00	0.00	0.00
12	*t2*	LPC 17:1	-3.85	1.95	3.88	*0.049*	0.02	0.00	0.98
13	*t2*	PI 34:0	-7.20	3.24	4.93	*0.026*	0.00	0.00	0.43

A logistic regression was performed to ascertain the effects of HDL on delta troponin groups. All models are adjusting for HDL. **Abbreviation**: Wald, Wald chi-square test; B, intercept; S.E, standard error; Exp(B), exponentiation of the B coefficient

**Table S17: Clinical demographics for subgroup comparison based on major adverse cardiovascular event (MACE)**(Related to Fig.4E)			
	No MACE (n=67)	MACE (n=13)	*p value*
Age(years)	63.01 ± 12.28	67.30 ± 11.62	0.249
Male sex (%)	44 (65.7)	10 (76.9)	0.428
LVEF (%)	61 (55, 70)	35 (34.25, 43.75)	<0.001
Body mass index (kg/m^2)	28 (24.55, 32.59)	23.70 (21.82, 30.84)	0.077
**Comorbidity (%)**			
Hypertension	32 (47.8)	5 (38.5)	0.538
Diabetes mellitus	15 (22.4)	1 (7.7)	0.225
Current smoker	15 (22.4)	7 (53.8)	0.02
Dyslipidemia	33 (49.3)	9 (69.2)	0.187
Hx of CAD	9 (13.4)	2 (15.4)	0.852
**Laboratory data**			
Triglyceride (mmol/l)	1.4 (1.0, 2.22)	1.0 (0.8, 1.47)	0.133
Cholesterol (mmol/l)	4.85 (4.1, 5.42)	4.3 (2.95, 5.12)	0.225
HDL cholesterol (mmol/l)	1.1 (0.9, 1.30)	1.3 (0.77, 1.57)	0.351
LDL cholesterol (mmol/l)	2.8 (2.02, 3.47)	2.4 (1.25, 3.15)	0.27
Creatinine (mmol/l)	89 (71, 104)	86 (64, 118)	0.819
**Medications at baseline (%)**			
ASA	13 (19.4)	5 (38.5)	0.132
ACEI/ARB	16 (23.9)	4 (30.8)	0.6
Beta blocker	5 (7.5)	3 (23.1)	0.086
Statin	15 (22.4)	5 (38.5)	0.221
**Additional parameters**			
Minutes from the onset of chest pain to reperfusion	146 (86, 99)	166 (250, 217)	0.896
Peak CK (Units/L)	906 (373.5, 1516.5)	3450 (352.0, 5836.5)	0.036
Peak TnT (ng/L)	1889 (994, 3867)	5813 (1997, 10000)	0.016
**Culprit vessel (%)**			
LAD Infarct (%)	27 (40.3)	9 (69.2)	0.055
RCA Infarct (%)	33 (49.3)	4 (30.8)	0.221
Circumflex Infarct (%)	10 (14.9)	0 (0)	0.136

Values are reported as mean ± standard deviation (SD), median (25th, 75th percentiles), or count (percentage) as applicable. The Chi-square test was used for categorical variables, while Student's t-test or Mann-Whitney U test was used for continuous variables to assess for statistical significance across sample groups as applicable based on data distribution. Abbreviations: LVEF = left ventricular ejection fraction; Hx of CAD = history of coronary artery disease; HDL = high-density lipoprotein; LDL = low-density lipoprotein; ASA = Acetylsalicylic acid; ACEI = Angiotensin-converting enzyme (ACE) inhibitors; ARB = Angiotensin II receptor blockers;

CK = Creatine kinase; TnT = troponin T; LAD = Left anterior descending coronary artery; RCA = Right coronary artery.
